# Design, Synthesis and Biological Evaluation of [1,2,4]Triazolo[1,5-*a*]pyrimidine Indole Derivatives against Gastric Cancer Cells MGC-803 via the Suppression of ERK Signaling Pathway

**DOI:** 10.3390/molecules27154996

**Published:** 2022-08-05

**Authors:** Guang-Xi Yu, Ying Hu, Wei-Xin Zhang, Xin-Yi Tian, Sai-Yang Zhang, Yan Zhang, Shuo Yuan, Jian Song

**Affiliations:** 1 School of Basic Medical Sciences, Zhengzhou University, Zhengzhou 450001, China; 2 Guana’anmen Hospital, China Academy of Chinese Medicinal Sciences, Beijing 100053, China; 3 Key Laboratory of Advanced Drug Preparation Technologies (Ministry of Education), Institute of Drug Discovery & Development, School of Pharmaceutical Sciences, Zhengzhou University, Zhengzhou 450001, China; 4 Children’s Hospital Affiliated of Zhengzhou University, Henan Children’s Hospital, Zhengzhou Children’s Hospital, Zhengzhou 450018, China

**Keywords:** [1,2,4]triazolo[1,5-*a*]pyrimidine, indole, antiproliferative activities, ERK signaling pathway

## Abstract

[1,2,4]Triazolo[1,5-*a*]pyrimidine and indole skeletons are widely used to design anticancer agents. Therefore, in this work, a series of [1,2,4]triazolo[1,5-*a*]pyrimidine indole derivatives were designed and synthesized by the molecular hybridization strategy. The antiproliferative activities of the target compounds **H1**–**H18** against three human cancer cell lines, MGC-803, HCT-116 and MCF-7, were tested. Among them, compound **H12** exhibited the most active antiproliferative activities against MGC-803, HCT-116 and MCF-7 cells, with IC_50_ values of 9.47, 9.58 and 13.1 μM, respectively, which were more potent than that of the positive drug **5-Fu**. In addition, compound **H12** could dose-dependently inhibit the growth and colony formation of MGC-803 cells. Compound **H12** exhibited significant inhibitory effects on the ERK signaling pathway, resulting in the decreased phosphorylation levels of ERK1/2, c-Raf, MEK1/2 and AKT. Furthermore, compound **12** induced cell apoptosis and G2/M phase arrest, and regulated cell cycle-related and apoptosis-related proteins in MGC-803 cells. Taken together, we report here that [1,2,4]triazolo[1,5-*a*]pyrimidine indole derivatives, used as anticancer agents via the suppression of ERK signaling pathway and the most active compound, **H12**, might be a valuable hit compound for the development of anticancer agents.

## 1. Introduction

**Figure 1:**
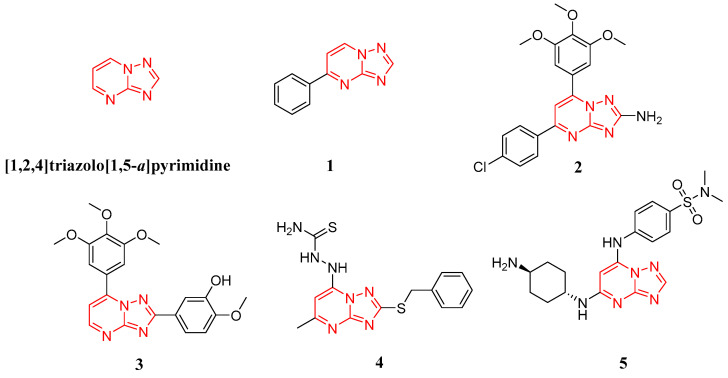
Structures of [1,2,4]triazolo[1,5-*a*]pyrimidine and its derivates as anticancer agents.

[1,2,4]Triazolo[1,5-*a*]pyrimidine, as an important class of bicyclic *N*-heteroarenes, exhibits versatile bioactivities, such as antibacterial [1,2,3], antiviral [4,5,6] and anticancer activities [7,8,9,10,11,12]. Recently, [1,2,4]triazolo[1,5-*a*]pyrimidine derivatives as anticancer agents via acting on different targets, such as tubulin [9,10,11,13,14], LSD1 [15,16] and CDK2 [17], have aroused remarkable research attention and exhibited potent antitumor activities. 5-Phenyl-[1,2,4]triazolo[1,5-*a*]pyrimidine **1** as a cytotoxic agent effectively inhibited the growth of MCF-7 cells, with an IC_50_ value of 3.91 μM [7]. As a tubulin polymerization inhibitor, compound **2** showed significant antiproliferative activity against HCT-116 cells, with an IC_50_ value of 0.53 μM, and compound **2** could induce cell apoptosis and G2/M phase arrest in HCT-116 cells [9]. Compound **3** could potently inhibit the polymerization of tubulin (IC_50_ = 3.84 μM) and display significant inhibitory potency on T47D, HCT29 and A549 cells (IC_50_ = 3.49, 0.24 and 6.05 μM, respectively) [11]. Compound 4 was identified as a potent LSD1/KDM1A inhibitor (IC_50_ = 0.154 μM), with potent inhibition toward MGC-803 and PC9 cells (IC_50_ = 2.1 and 12.4 μM, respectively)[16]. [1,2,4]Triazolo[1,5-*a*]pyrimidine derivative **5** [17] showed potent and selective inhibitory effect on CDK2 (IC_50_ = 0.12 μM), which was 167 times stronger than GSK-3β (Figure 1).

**Figure 2:**
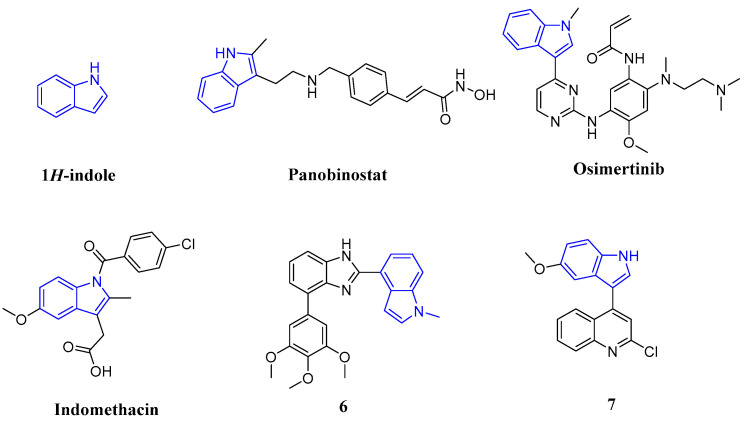
Structures of indole and its previously reported derivates.

Indoles, some of the most promising *N*-containing heterocycles, possess a wide range of biological activities [18,19,20,21,22,23,24]. There are many drugs containing indoles that have been approved by the FDA for the treatment of different human diseases, such as the anticancer drugs **Panobinostat** (HDAC inhibitor) [25] and **Osimertinib** (3rd EGFR tyrosine kinase inhibitor) [26], and the anti-inflammatory drug **Indomethacin** [27]. Therefore, indoles are widely considered as one class of dominant skeletons for the development of novel drugs [18,22,28,29,30]. In addition, indole biaryl structures have been widely used in the discovery of antitumor drugs. For example, **Osimertinib,** containing the indole-pyrimidine core structure, was approved for the treatment of non-small-cell lung cancer [26]. Indazole-benzimidazole derivative **6** [28], as a tubulin polymerization inhibitor, exhibited potent inhibitory activities against four cancer cell lines (MCF-7, A549, Hela and B16-F10 cells) and one paclitaxel-resistant cancer cell line (A2780/T cells) at low nanomolar levels. Quinoline-indole derivative **7** [29] exhibited potent inhibitory potency on MGC-803, HCT-116 and KYSE450 cells (IC_50_ = 0.58, 0.68 and 0.59 μM, respectively), and induced cell apoptosis and G2/M phase arrest (Figure 2).

**Figure 3:**
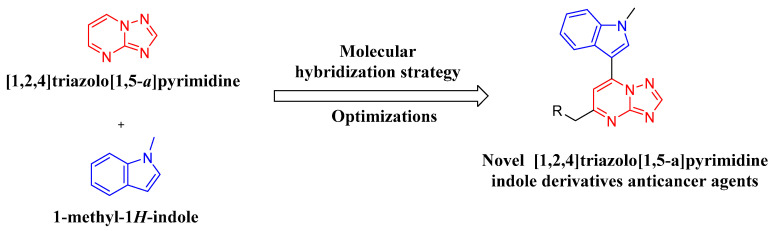
Design of [1,2,4]triazolo[1,5-*a*]pyrimidine indole derivatives.

Considering the important roles of [1,2,4]triazolo[1,5-*a*]pyrimidine and indole skeletons in the development of anticancer agents, we linked an indole fragment to the [1,2,4]triazolo[1,5-*a*]pyrimidine scaffold through the principle of molecular hybridization strategy, and then introduced amino fragments or benzothiazole groups to obtain novel [1,2,4]triazolo[1,5-*a*]pyrimidine indole derivatives (Figure 3). Although surgery procedures and chemotherapy have improved greatly in recent years, it is highly desirable to develop newly targeted therapy for human cancers with minimal side-effects and new classes of anti-cancer agents with excellent selectivity between cancer and normal cells. Therefore, the antiproliferative activities of target compounds against three human cancer cell lines, MGC-803, HCT-116 and MCF-7, were tested, and the potential antitumor mechanisms of the most active compound were further explored.

## 2. Results and Discussion

### 2.1. Chemistry

**Scheme 1:**
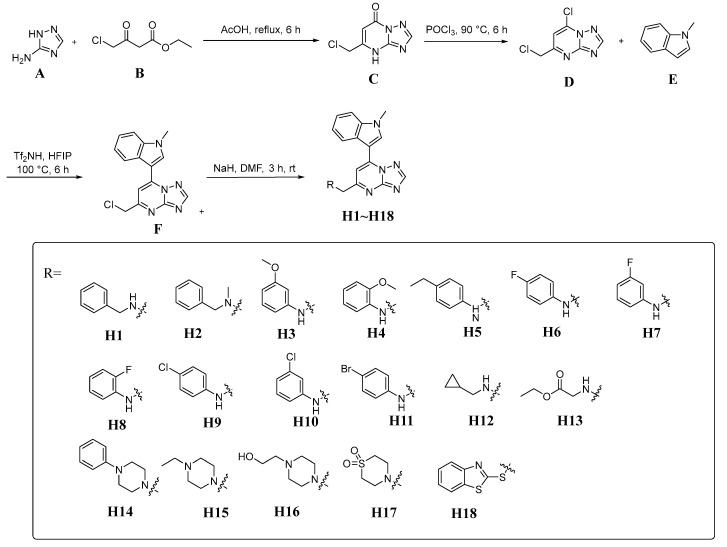
Synthesis of the target compounds **H1**–**H18.**

As shown in Scheme 1, the target compounds **H1**–**H18** were synthesized from the commercially available compound 1*H*-1,2,4-triazol-5-amine (**A**) and ethyl 4-chloro-3-oxobutanoate (**B**) in four steps. The cyclization of *H*-1,2,4-triazol-5-amine (**A**) and ethyl 4-chloro-3-oxobutanoate (**B**) in acetic acid (AcOH) generated compound **C**, which was then converted to 7-chloro-5-(chloromethyl)-[1,2,4]triazolo[1,5-*a*]pyrimidine (**D**) in phosphorus oxychloride (POCl_3_). Compound **C** reacted with 1-methyl-1*H*-indole (**E**) to afford compound **F** in the presence of Bis(trifluoromethane sulfonimide) (Tf_2_NH) in hexafluoroisopropanol (HFIP). The substitution reaction of compound **F** with substituted amines or 2-mercaptobenzothiazole resulted in the target compounds **H1**–**H18**. Characterization of target compounds **H1**–**H18** was realized by means of the NMR and HRMS spectra (Supplementary Materials Figures S1–S54).

### 2.2. Biological Evaluation

#### Antiproliferative Activities of Compounds **H1**–**H18**

The MTT assay was carried out to explore the in vitro antiproliferative activities of compounds **H1**–**H18** against MGC-803 cells (human gastric cancer cells), HCT-116 cells (human colorectal carcinoma cells) and MCF-7 cells (human breast cancer cells) using **5-Fu** as the positive drug. The following Table 1 summarized the results of the antiproliferative activities of compounds **H1**–**H18**.

As shown in Table 1, among the compounds **H1**–**H18**, compound **H12** exhibited the most active antiproliferative activities against MGC-803, HCT-116 and MCF-7 cells, with IC_50_ values of 9.47, 9.58 and 13.1 μM, respectively, which were more potent than that of the positive drug **5-Fu**. On the whole, most of compounds displayed moderate antiproliferative activities against three cancer cells, with IC_50_ values < 80 μM. The antiproliferative activities of compounds **H1**–**H18** varied with its substituent groups of R. Additionally, most of compounds were more sensitive to HCT cells than MGC-803 and MCT-cells, excepting compounds **H5**, **H9**, **H12** and **H18**. As for compounds **H3**–**H11**, the inhibitory activities varied with its substituent groups at phenyl groups of R. When the para position at phenyl of R were electron donating group ethyl substituent group (compound **H5**), its activity against HCT-116 cells was better than the para position at phenyl of R with electron withdrawing groups F (compound **H6**), Cl (compound **H9**) and Br (compound **H11**). In addition, the relationships between the electron withdrawing groups and the antiproliferative activities against HCT-116 cells were 4-Cl > 3-F > 4-Br > 3-Cl > 2-F > 4-F. Compared to compounds **H3**–**H11**, the change of arylamines to cyclopropylmethanamine (compound **H12**) improved inhibitory efficacy on MGC-803, HCT-116 and MCF-7 cells, indicating the appropriate volume of substituents of R is conducive to the maintenance of the activities. However, the introduction of six membered nitrogen-containing heterocyclic groups or the benzothiazole group of R had little effect on the activities, compared with compounds **H5** and **H12**.

### 2.3. Inhibitory Effects of Compound **H12** on MGC-803 Cells

**Figure 4:**
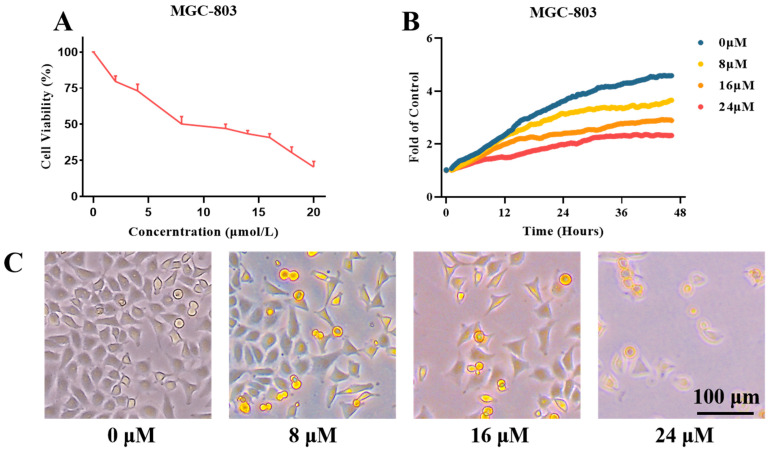
Effects of compound **H12** on MGC-803 cells. MGC-803 cells were treated with indicated concentrations of compound **H12** for 48 h. (**A**) Cell viability of MGC-803 cells after treatment with DMSO or compound **H12**. The experiment was repeated three times independently. (**B**) Cell growth curves of MGC-803 cells after treatment with DMSO or compound **H12**. (**C**) The morphological changes of MGC-803 cells after treatment with DMSO or compound **H12**.

To illustrate the inhibitory effect of compound **H12** on gastric cancer cells MGC-803, MGC-803 cells were treated with different concentrations of compound **H12** and then detected by MTT assay. As shown in Figure 4A,B, compound **H12** could inhibit MGC-803 cells in dose- and time-dependent manners. Next, the growth status of MGC-803 under the effects of compound **H12** were monitored by Real Time Cell Analysis (RTCA). The results are shown in Figure 4C: compound **H12** at different concentrations inhibited cell growth to varying degrees. The morphological changes of the MGC-803 cells after treatment with compound **H12** could be observed, and it was found that compound **H12** caused a decrease in the density and cell rupture in MGC-803 cells. These results indicated that compound **H12** could dose- and time-dependently inhibit gastric cancer cells MGC-803.

### 2.4. Inhibition of the ERK Signaling Pathway by Compound **H12**

**Figure 5:**
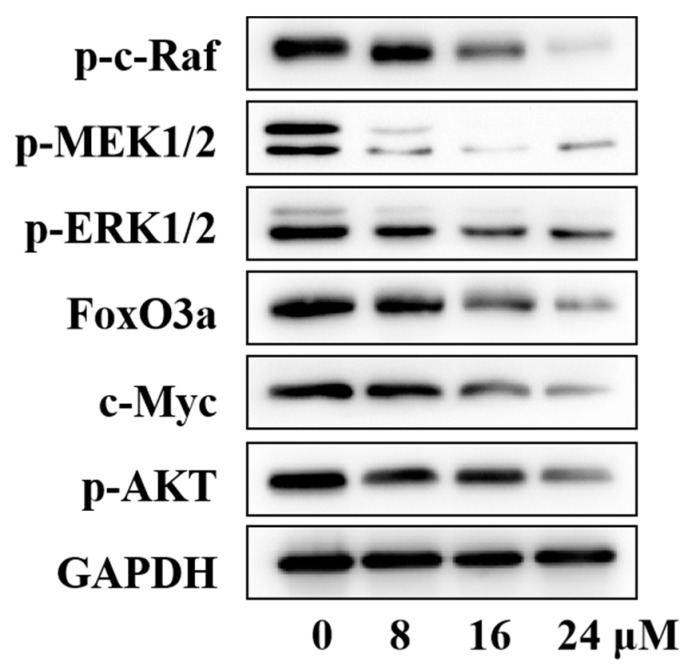
Inhibitory effects of compound **H12** on MGC-803 cells. MGC-803 cells were treated with indicated concentrations of compound **H12** for 48 h. The cell lysates were collected for Western blotting with the indicated antibodies.

The ERK signaling pathway plays an important role in regulating cell growth and is highly activated in cancers, and, therefore, has been identified as a promising therapeutic target for the treatment of human cancers [31,32]. Through the screening of various signaling pathways, we found that compound **H12** exhibited significant inhibitory effects on the ERK signaling pathway. As shown in Figure 5, the activation (phosphorylation) levels of ERK1/2, the core protein of the ERK signaling pathway, and its upstream proteins c-Raf and MEK1/2, were significantly inhibited in MGC-803 cells in a dose-dependent manner after treatment with compound **H12**. The levels of FoxO3 and c-Myc, which are regulated by the ERK signaling pathway [33], were also significantly down-regulated. In addition, compound **H12** could effectively reduce the phosphorylation level of AKT, indicating that the AKT signaling pathway, a related pathway to the ERK signaling pathway, was also inhibited. The above results suggested that compound **H12** might be an effective inhibitor of the ERK signaling pathway.

### 2.5. Effects of Compound ***H11*** on the Proliferation of MGC-803 Cells

**Figure 6:**
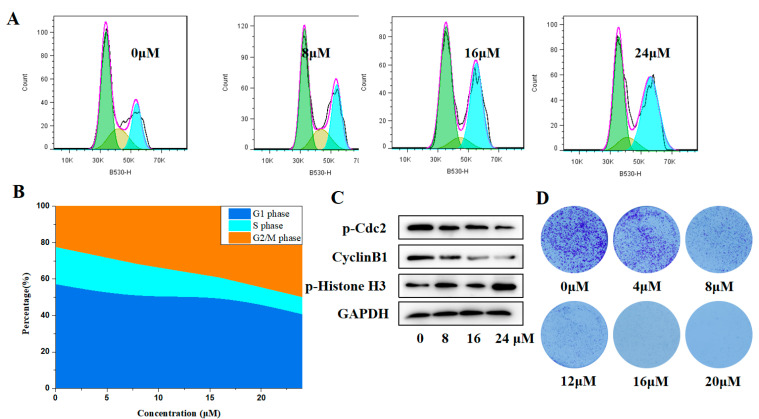
Effects of compound **H12** on cell proliferation. For A–C, MGC-803 cells were treated with indicated concentrations of compound **H12** for 48 h. For D, MGC-803 cells were treated with indicated concentrations of compound **H12** for 7 days. (**A**,**B**) Cell cycle distribution of treated MGC-803 cells. (**C**) Levels of cell cycle-related proteins in treated MGC-803 cells. (**D**) The colonies that formatted after treatment with compound **H12** in MGC-803 cells.

The ERK signaling pathway could regulate cell proliferation [34]. Therefore, the effects of compound **H12** on cell proliferation were next examined. As shown in Figure 6B, the percentage of the G2/M phase of MGC-803 cells was concentration-dependently increased after treatment with compound **H12**. At concentrations of 8, 16 and 24 μM, the percentages of G2/M phase induced by compound **H12** were 31.88, 39.32 and 50%, respectively. Meanwhile, the percentages of G2/M phase in the control group were 22.52%. The effects of compound **H12** on the levels of cycle-related proteins were explored using Western blotting assay. As shown in Figure 6C, the levels of cell cycle-related proteins p-Cdc2 and CyclinB1 decreased and the levels of M-phase marker protein p-Histone H3 increased after treatment with compound **H12** in a dose-dependent manner. The decrease in cell proliferation capacity was also manifested in the cell colony formatting activity (Figure 6D). The results of the colony formatting assay suggested that a low concentration of compound **H12** could significantly inhibit the colony formatting activity of MGC-803 cells. The above results indicated compound **H12** had significant inhibitory effects on the cell proliferation of MGC-803 cells.

### 2.6. Effects of Compound ***H12*** on the Apoptosis of MGC-803 Cells

**Figure 7:**
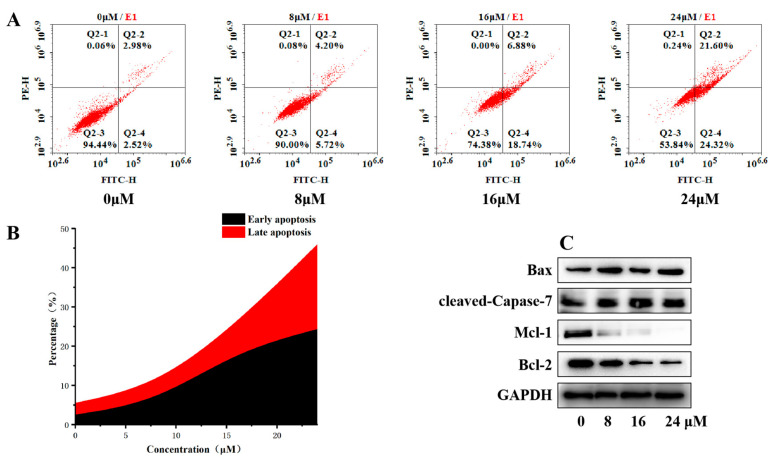
Effects of compound **H12** on cell apoptosis. MGC-803 cells were treated with indicated concentrations of compound **H12** for 48 h. (**A**,**B**) Apoptosis rates of treated MGC-803 cells; (**C**) level of apoptosis related proteins in treated MGC-803 cells.

The ERK signaling pathway also affects apoptosis in cancer cells [35]. Therefore, the effects of compound **H12** on cell apoptosis were next explored using the flow cytometry assay. As shown in Figure 7A,B, the percentage of apoptotic cells in MGC-803 cells increased after treatment with compound **H12** for 48 h. At concentrations of 8, 16 and 24 μM, the percentages of apoptotic cells induced by compound **H12** were 9.92, 25.62 and 45.92%, respectively. Meanwhile, the percentages of apoptotic cells in the control group were 5.50%. We also used the Western blotting assay to explore the effects of compound **H12** on the levels of apoptosis-related proteins. As shown in Figure 7C, apoptosis-like changes were also observed at the protein levels. The level of the pro-apoptotic protein Bax was increased and the levels of the anti-apoptotic proteins Mcl-1 and Bcl-2 were decreased. In addition, compound **H12** could up-regulate the level of cleaved-Caspase7. In conclusion, compound **H12** could induce cell apoptosis and regulate apoptosis-related proteins in MGC-803 cells.

## 3. Conclusions

In conclusion, [1,2,4]triazolo[1,5-*a*]pyrimidine indole derivatives were designed and synthesized by the molecular hybridization strategy and their antiproliferative activities against three human cancer cell lines, MGC-803, HCT-116 and MCF-7, were tested. Among these compounds, compound **H12** exhibited the most active antiproliferative activities against MGC-803, HCT-116 and MCF-7 cells, with IC_50_ values of 9.47, 9.58 and 13.1 μM, respectively, which were more potent than that of the positive drug **5-Fu**. Further antitumor mechanisms suggested that compound **H12** dose-dependently inhibited the growth and colony formation of MGC-803 cells. After screening different signaling pathways, it was found that compound **H12** inhibited the activity of the ERK signaling pathway, resulting in decreased phosphorylation levels of ERK1/2, c-Raf, MEK1/2 and AKT. The ERK signaling pathway is closely related to both cell proliferation and apoptosis. Therefore, it could be found that compound **H12** could arrest gastric cancer cells MGC-803 in the G2/M phase and induce apoptosis by regulating related proteins. In conclusion, compound **H12** could inhibit the ERK signaling pathway, and inhibit the proliferation of and induce apoptosis in MGC-803 cells.

## 4. Materials and Methods

All the chemical reagents were purchased from commercial suppliers (Energy chemical Company and Aladdin reagent, Shanghai, China). NMR and HRMS spectral data were recorded with a Bruker spectrometer (Karlsruhe, Baden-Wurttemberg, Germany).

### 4.1. Synthesis of Compound ***C***

A solution of commercially available compound 1*H*-1,2,4-triazol-5-amine (**A**) (1.0 mmol, 1.0 eq) and ethyl 4-chloro-3-oxobutanoate (**B**) (1.0 mmol, 1.2 eq) were added into 20 mL acetic acid at 25 °C. Then, the reaction mixture was stirred at 120 °C for 6 h. After 8 h, the reaction mixture was evaporated to give crude products and 20 mL ethyl acetate was added, giving a white solid. The white solid was filtered and dried (**C**) without further purification.

### 4.2. Synthesis of Compound ***D***

A solution of compound ***C*** (1.0 mmol, 1.0 eq) was added into 20 mL phosphorus oxychloride at 25 °C. Then, the reaction mixture was stirred at 90 °C for 6 h. After 6 h, the reaction mixture was evaporated to give crude product, and then crude product was purified to give compound **D** by column chromatography.

### 4.3. Synthesis of Compound ***F***

A solution of compound **C** (1.0 mmol, 1.1 eq), 1-methyl-1*H*-indole (**E**) (1.0 mmol, 1.0 eq) and Bis(trifluoromethane sulfonimide) (0.2 mmol, 0.2 eq) was added into 20 mL HIFP at 25 °C. Then, the reaction mixture was stirred at 100 °C for 6 h. After 6 h, the reaction mixture was evaporated to give crude product, and then crude product was purified to give compound **F** by column chromatography.

### 4.4. Synthesis of Compounds ***H1***–***H18***

A solution of compound **F** (1.0 mmol, 1.1 eq), 1 substituted amines/2-mercaptobenzothiazole (**E**) (1.0 mmol, 1.0 eq) and NaH (0.2 mmol, 0.2 eq) was added into 10 mL DMF. Then, the reaction mixture was stirred at 25 °C for 3 h. After 3 h, the reaction mixture was added to 10 mL H_2_O and then extracted three times using ethyl acetate. The organic phases were combined and dried with anhydrous magnesium sulfate, which then were evaporated to give crude products. The products were purified to obtain compounds **H1**–**H18** by column chromatography.

*N*-benzyl-1-(7-(1-methyl-1*H*-indol-3-yl)-[1,2,4]triazolo[1,5-*a*]pyrimidin-5-yl)methanamine (**H1**).

White powdery solid, Yield, 66%, M.p. 154–155 °C.^1^H NMR (400 MHz, DMSO-*d_6_*) δ 9.07 (s, 1H), 8.70 (s, 1H), 8.18 (d, *J* = 7.9 Hz, 1H), 7.94 (s, 1H), 7.69 (d, *J* = 8.0 Hz, 1H), 7.41 (t, *J* = 8.6 Hz, 3H), 7.33 (d, *J* = 7.4 Hz, 3H), 7.25 (t, *J* = 7.2 Hz, 1H), 4.02 (s, 2H), 4.00 (s, 3H), 3.85 (s, 2H). ^13^C NMR (100 MHz, DMSO-*d_6_*) δ 193.24, 155.29, 142.61, 137.63, 137.22, 134.56, 129.45, 129.23, 129.12, 128.45, 125.00, 123.23, 122.12, 120.22, 111.44, 104.17, 102.82, 63.92, 51.09, 33.45. HRMS: calcd for C_22_H_21_N_6_ [M + H]^+^, 369.1822, found: 369.1823.

*N*-benzyl-N-methyl-1-(7-(1-methyl-1*H*-indol-3-yl)-[1,2,4]triazolo[1,5-*a*] pyrimidin-5-yl)methanamine (**H2**).

White powdery solid, Yield, 61%, M.p. 144–146 °C. ^1^H NMR (400 MHz, DMSO-*d_6_*) δ 9.05 (s, 1H), 8.70 (s, 1H), 8.07 (d, *J* = 7.6 Hz, 1H), 7.91 (s, 1H), 7.70 (d, *J* = 6.9 Hz, 1H), 7.46 (d, *J* = 7.4 Hz, 2H), 7.42 (d, *J* = 2.7 Hz, 2H), 7.37 (t, *J* = 7.3 Hz, 2H), 7.28 (t, *J* = 7.2 Hz, 1H), 4.00 (s, 3H), 3.82 (s, 2H), 3.70 (s, 2H), 2.30 (s, 3H). ^13^C NMR (100 MHz, DMSO-*d_6_*) δ 165.40, 155.42, 155.07, 142.35, 138.82, 137.23, 137.14, 128.62, 128.24, 127.05, 125.04, 123.11, 122.07, 119.54, 111.55, 104.22, 102.90, 62.42, 61.10, 42.26, 33.38. HRMS: calcd for C_23_H_23_N_7_ [M + H]^+^, 383.1979, found: 383.1979.

2-Methoxy-*N*-((7-(1-methyl-1*H*-indol-3-yl)-[1,2,4]triazolo[1,5-*a*]pyrimidin-5-yl)methyl)aniline (**H3**).

White powdery solid, Yield, 58%, M.p. 167–168 °C. ^1^H NMR (400 MHz, CDCl_3_) δ 8.90 (s, 1H), 8.52 (s, 1H), 7.65–7.58 (m, 2H), 7.43 (d, *J* = 8.1 Hz, 1H), 7.35 (t, *J* = 7.6 Hz, 1H), 7.22 (t, *J* = 7.6 Hz, 1H), 6.85 (dd, *J* = 20.2, 7.8 Hz, 2H), 6.74 (t, *J* = 7.5 Hz, 1H), 6.63 (d, *J* = 7.7 Hz, 1H), 5.29 (s, 1H), 4.69 (s, 2H), 3.93 (s, 6H). ^13^C NMR (100 MHz, CDCl_3_) δ 165.50, 155.21, 147.12, 143.19, 137.42, 137.13, 136.73, 125.84, 123.34, 122.28, 121.39, 120.10, 117.45, 110.55, 110.52, 109.79, 104.24, 103.82, 55.60, 49.16, 33.74. HRMS: calcd for C_20_H_23_N_6_O [M + H]^+^, 385.1771, found: 385.1771.

3-Methoxy-*N*-((7-(1-methyl-1*H*-indol-3-yl)-[1,2,4]triazolo[1,5-*a*]pyrimidin-5-yl)methyl)aniline (**H4**).

White powdery solid, Yield, 65%, M.p. 159–160 °C. ^1^H NMR (400 MHz, CDCl_3_) δ 8.90 (s, 1H), 8.52 (s, 1H), 7.72 (d, *J* = 8.0 Hz, 1H), 7.60 (s, 1H), 7.45 (d, *J* = 8.1 Hz, 1H), 7.37 (t, *J* = 7.6 Hz, 1H), 7.26 (s, 1H), 7.13 (t, *J* = 8.0 Hz, 1H), 6.35 (dd, *J* = 16.6, 7.4 Hz, 3H), 4.97 (s, 1H), 4.62 (s, 2H), 3.93 (s, 3H), 3.77 (s, 3H). ^13^C NMR (100 MHz, CDCl_3_) δ 164.41, 161.03, 155.17, 148.70, 143.14, 137.44, 136.86, 130.27, 125.78, 123.44, 122.40, 120.09, 110.61, 106.15, 104.11, 103.88, 103.39, 99.30, 55.16, 49.08, 33.76. HRMS: calcd for C_20_H_23_N_6_O [M + H]^+^, 385.1771, found: 385.1771.

4-Ethyl-*N*-((7-(1-methyl-1*H*-indol-3-yl)-[1,2,4]triazolo[1,5-*a*]pyrimidin-5-yl)methyl)aniline (**H5**).

White powdery solid, Yield, 56%, M.p. 156–157 °C. ^1^H NMR (400 MHz, DMSO-*d_6_*) δ 9.00 (d, *J* = 13.2 Hz, 1H), 8.70 (d, *J* = 8.0 Hz, 1H), 7.72—7.63 (m, 3H), 7.36 (t, *J* = 7.6 Hz, 1H), 7.20 (t, *J* = 7.5 Hz, 1H), 6.96 (d, *J* = 7.9 Hz, 2H), 6.66 (d, *J* = 7.9 Hz, 2H), 4.57 (s, 2H), 3.96 (s, 3H), 2.43 (dd, *J* = 14.8, 7.4 Hz, 2H), 1.08 (t, *J* = 7.5 Hz, 3H). 13C NMR (100 MHz, DMSO-*d_6_*) δ 166.54, 155.49, 155.17, 145.81, 142.15, 137.13, 131.68, 128.72, 128.31, 124.96, 123.02, 121.93, 121.82, 119.69, 112.63, 111.34, 103.41, 102.79, 48.42, 33.34, 27.26, 15.96. HRMS: calcd for C_23_H_23_N_6_ [M + H]^+^, 383.1979, found: 383.1979.

4-Fluoro-*N*-((7-(1-methyl-1*H*-indol-3-yl)-[1,2,4]triazolo[1,5-*a*]pyrimidin-5-yl)methyl)aniline (**H6**).

White powdery solid, Yield, 54%, M.p. 156–157 °C. ^1^H NMR (400 MHz, DMSO-*d_6_*) δ 9.04 (s, 1H), 8.71 (s, 1H), 7.76 (d, *J* = 8.1 Hz, 1H), 7.72 (s, 1H), 7.66 (d, *J* = 8.2 Hz, 1H), 7.37 (t, *J* = 7.6 Hz, 1H), 7.24 (t, *J* = 7.5 Hz, 1H), 6.96 (t, *J* = 8.6 Hz, 2H), 6.72 (dd, *J* = 7.7, 4.0 Hz, 2H), 6.62 (t, *J* = 6.0 Hz, 1H), 4.58 (d, *J* = 6.1 Hz, 2H), 3.98 (s, 3H). ^13^C NMR (100 MHz, DMSO-*d_6_*) δ 166.15, 155.75, 155.51, 155.22, 153.45, 144.71, 142.23, 137.23, 137.16, 124.96, 123.08, 121.96, 119.64, 115.56, 115.34, 113.33, 113.26, 111.44, 103.36, 102.77, 48.60, 33.38. HRMS: calcd for C_21_H_18_FN_6_ [M + H]^+^, 373.1571, found: 373.1573.

3-Fluoro-*N*-((7-(1-methyl-1*H*-indol-3-yl)-[1,2,4]triazolo[1,5-*a*]pyrimidin-5-yl)methyl)aniline (**H7**).

White powdery solid, Yield, 60%, M.p. 150–151 °C. ^1^H NMR (400 MHz, DMSO-*d_6_*) δ 9.05 (s, 1H), 8.72 (s, 1H), 7.81 (d, *J* = 8.1 Hz, 1H), 7.72 (s, 1H), 7.67 (d, *J* = 8.2 Hz, 1H), 7.38 (t, *J* = 7.6 Hz, 1H), 7.25 (t, *J* = 7.5 Hz, 1H), 7.12 (q, *J* = 7.7 Hz, 1H), 6.98 (t, *J* = 6.1 Hz, 1H), 6.56 (dd, *J* = 15.2, 10.6 Hz, 2H), 6.36 (t, *J* = 8.4 Hz, 1H), 4.63 (d, *J* = 6.3 Hz, 2H), 3.98 (s, 3H). ^13^C NMR (100 MHz, DMSO-*d_6_*) δ 165.64, 164.71, 162.32, 155.50, 155.23, 150.31, 150.20, 142.29, 137.28, 137.18, 130.56, 130.45, 124.96, 123.09, 121.99, 119.65, 111.44, 108.79, 103.35, 102.77, 102.57, 102.36, 98.97, 98.72, 48.00, 33.38. HRMS: calcd for C_21_H_18_FN_6_ [M + H]^+^, 373.1571, found: 373.1572.

2-Fluoro-*N*-((7-(1-methyl-1*H*-indol-3-yl)-[1,2,4]triazolo[1,5-*a*]pyrimidin-5-yl)methyl)aniline (**H8**).

White powdery solid, Yield, 49%, M.p. 158–159 °C. ^1^H NMR (400 MHz, DMSO-*d_6_*) δ 9.05 (s, 1H), 8.72 (s, 1H), 7.81 (d, *J* = 8.1 Hz, 1H), 7.72 (s, 1H), 7.67 (d, *J* = 8.2 Hz, 1H), 7.38 (t, *J* = 7.6 Hz, 1H), 7.25 (t, *J* = 7.5 Hz, 1H), 7.12 (q, *J* = 7.7 Hz, 1H), 6.98 (t, *J* = 6.1 Hz, 1H), 6.56 (dd, *J* = 15.2, 10.6 Hz, 2H), 6.36 (t, *J* = 8.4 Hz, 1H), 4.63 (d, *J* = 6.3 Hz, 2H), 3.98 (s, 3H). ^13^C NMR (100 MHz, DMSO-*d_6_*) δ 165.64, 164.71, 162.32, 155.50, 155.23, 150.31, 150.20, 142.29, 137.28, 137.18, 130.56, 130.45, 124.96, 123.09, 121.99, 119.65, 111.44, 108.79, 103.35, 102.77, 102.57, 102.36, 98.97, 98.72, 48.00, 33.38. HRMS: calcd for C_21_H_18_FN_6_ [M + H]^+^, 373.1571, found: 373.1571.

4-Chloro-*N*-((7-(1-methyl-1*H*-indol-3-yl)-[1,2,4]triazolo[1,5-*a*]pyrimidin-5-yl)methyl)aniline (**H9**).

White powdery solid, Yield, 48%, M.p. 155–156 °C. ^1^H NMR (400 MHz, DMSO-*d_6_*) δ 9.05 (s, 1H), 8.72 (s, 1H), 7.75 (d, *J* = 8.0 Hz, 1H), 7.71–7.58 (m, 2H), 7.38 (t, *J* = 7.3 Hz, 1H), 7.24 (t, *J* = 7.4 Hz, 1H), 7.14 (d, *J* = 8.3 Hz, 2H), 6.88 (t, *J* = 5.2 Hz, 1H), 6.74 (d, *J* = 8.2 Hz, 2H), 4.61 (d, *J* = 5.7 Hz, 2H), 3.99 (s, 3H). ^13^C NMR (100 MHz, DMSO-*d_6_*) δ 165.81, 155.26, 147.03, 142.29, 137.27, 137.19, 128.78, 124.97, 123.11, 121.97, 119.73, 119.64, 113.94, 111.47, 103.33, 102.76, 48.11, 33.39. HRMS: calcd for C_21_H_18_ClN_6_ [M + H]^+^, 389.1276, found: 389.1276.

3-Chloro-*N*-((7-(1-methyl-1*H*-indol-3-yl)-[1,2,4]triazolo[1,5-*a*]pyrimidin-5-yl)methyl)aniline (**H10**).

White powdery solid, Yield, 55%, M.p. 152–153 °C. ^1^H NMR (400 MHz, DMSO-*d_6_*) δ 9.06 (s, 1H), 8.72 (s, 1H), 7.81 (d, *J* = 8.1 Hz, 1H), 7.72 (s, 1H), 7.68 (d, *J* = 8.3 Hz, 1H), 7.39 (t, *J* = 7.5 Hz, 1H), 7.26 (t, *J* = 7.7 Hz, 1H), 7.11 (t, *J* = 8.1 Hz, 1H), 6.97 (t, *J* = 6.4 Hz, 1H), 6.80 (s, 1H), 6.69 (d, *J* = 8.1 Hz, 1H), 6.60 (d, *J* = 7.8 Hz, 1H), 4.63 (d, *J* = 6.3 Hz, 2H), 3.99 (s, 3H). ^13^C NMR (100 MHz, DMSO-*d_6_*) δ 165.56, 155.51, 155.26, 149.70, 142.30, 137.31, 137.20, 133.81, 130.60, 124.97, 123.12, 122.04, 119.67, 115.85, 111.80, 111.47, 111.18, 103.37, 102.76, 47.85, 33.40. HRMS: calcd for C_21_H_18_ClN_6_ [M + H]^+^, 389.1276, found: 389.1277.

4-Bromo-*N*-((7-(1-methyl-1*H*-indol-3-yl)-[1,2,4]triazolo[1,5-*a*]pyrimidin-5-yl)methyl)aniline (**H11**).

White powdery solid, Yield, 47%, M.p. 159–160 °C. ^1^H NMR (400 MHz, CDCl_3_) δ 8.98 (s, 1H), 8.55 (s, 1H), 7.78 (d, *J* = 8.0 Hz, 1H), 7.64 (s, 1H), 7.39 (ddd, *J* = 33.7, 21.1, 7.8 Hz, 7H), 6.65 (d, *J* = 8.5 Hz, 2H), 5.08 (s, 1H), 4.65 (s, 2H), 3.98 (s, 3H). ^13^C NMR (100 MHz, DMSO-*d_6_*) δ 165.75, 155.26, 147.39, 142.28, 137.28, 137.18, 131.60, 124.97, 123.11, 121.99, 119.63, 114.51, 111.48, 107.11, 103.31, 102.75, 48.01, 33.40. HRMS: calcd for C_21_H_18_BrN_6_ [M + H]^+^: 433.0771, found: 433.0773.

Cyclopropyl-*N*-((7-(1-methyl-1*H*-indol-3-yl)-[1,2,4]triazolo[1,5-*a*] pyrimidin-5-yl)methyl)methanamine (**H12**)

White powdery solid, Yield, 55%, M.p. 157–158 °C. ^1^H NMR (400 MHz, DMSO-*d_6_*) δ 9.04 (s, 1H), 8.68 (s, 1H), 8.19 (d, *J* = 7.4 Hz, 1H), 7.88 (s, 1H), 7.66 (d, *J* = 7.7 Hz, 1H), 7.38 (dd, *J* = 13.6, 6.8 Hz, 2H), 4.07 (s, 2H), 3.98 (s, 3H), 2.55 (d, *J* = 6.6 Hz, 2H), 1.01–0.92 (m, 1H), 0.43 (d, *J* = 7.2 Hz, 2H), 0.15 (d, *J* = 4.3 Hz, 2H). ^13^C NMR (100 MHz, DMSO-*d_6_*) δ 155.35, 155.12, 142.32, 137.34, 137.18, 125.03, 123.10, 122.03, 120.10, 111.37, 103.97, 102.92, 64.09, 52.92, 33.39, 11.36, 10.09. HRMS: calcd for C_19_H_21_N_6_ [M + H]^+^: 333.1822, found: 333.1823.

Ethyl((7-(1-methyl-1*H*-indol-3-yl)-[1,2,4]triazolo[1,5-*a*]pyrimidin-5-yl) methyl)glycinate (**H13**).

White powdery solid, Yield, 64%, M.p. 162–164 °C.^1^H NMR (400 MHz, DMSO-*d_6_*) δ 9.07 (s, 1H), 8.70 (s, 1H), 8.21 (d, *J* = 7.5 Hz, 1H), 7.89 (s, 1H), 7.68 (d, *J* = 7.7 Hz, 1H), 7.46–7.32 (m, 2H), 4.11 (dd, *J* = 14.3, 7.2 Hz, 4H), 4.00 (s, 3H), 3.54 (s, 2H), 1.19 (t, *J* = 6.9 Hz, 3H). ^13^C NMR (100 MHz, DMSO-*d_6_*) δ 171.43, 165.17, 155.36, 155.08, 142.31, 137.24, 137.17, 125.04, 123.08, 122.01, 120.12, 111.34, 104.06, 102.97, 60.17, 53.20, 49.43, 33.38, 14.04. HRMS: calcd for C_19_H_21_N_6_O_2_ [M + H]^+^, 365.1721, found: 365.1721.

7-(1-Methyl-1*H*-indol-3-yl)-5-((4-phenylpiperazin-1-yl)methyl)-[1,2,4] triazolo[1,5-*a*]pyrimidine (**H14**).

White powdery solid, Yield, 44%, M.p. 165–166 °C. ^1^H NMR (400 MHz, CDCl_3_) δ 8.99 (s, 1H), 8.54 (s, 1H), 8.11 (s, 1H), 8.01 (s, 1H), 7.49 (s, 1H), 7.40 (s, 2H), 7.27 (s, 2H), 6.96 (s, 2H), 6.87 (s, 1H), 3.97 (s, 3H), 3.90 (s, 2H), 3.31 (s, 4H), 2.82 (s, 4H). ^13^C NMR (101 MHz, CDCl_3_) δ 164.93, 156.14, 155.18, 151.23, 143.15, 137.50, 136.82, 129.15, 125.89, 123.45, 122.44, 120.06, 119.79, 116.05, 110.70, 104.85, 104.31, 64.12, 53.47, 49.33, 33.80. HRMS: calcd for C_25_H_26_N_7_ [M + H]^+^: 424.2244, found: 424.2246.

5-((4-Ethylpiperazin-1-yl)methyl)-7-(1-methyl-1*H*-indol-3-yl)-[1,2,4] triazolo[1,5-*a*]pyrimidine (**H15**).

White powdery solid, Yield, 66%, M.p. 163–165 °C.^1^H NMR (400 MHz, DMSO-*d_6_*) δ 9.04 (s, 1H), 8.70 (s, 1H), 8.09 (d, *J* = 7.3 Hz, 1H), 7.88 (s, 1H), 7.70 (d, *J* = 7.7 Hz, 1H), 7.50–7.30 (m, 2H), 4.01 (s, 3H), 3.78 (s, 2H), 2.59 (s, 4H), 2.38 (d, *J* = 6.9 Hz, 4H), 1.01 (t, *J* = 7.1 Hz, 3H). ^13^C NMR (100 MHz, DMSO-*d_6_*) δ 164.74, 155.48, 155.11, 142.32, 137.24, 137.10, 125.07, 123.10, 122.09, 119.58, 111.55, 104.38, 102.93, 63.05, 52.63, 52.49, 51.56, 33.38, 11.78. HRMS: calcd for C_21_H_26_N_7_ [M + H]^+^: 376.2244, found: 376.2244.

2-(4-((7-(1-Methyl-1*H*-indol-3-yl)-[1,2,4]triazolo[1,5-*a*]pyrimidin-5-yl)methyl)piperazin-1-yl)ethan-1-ol (**H16**).

White powdery solid, Yield, 55%, M.p. 157–158 °C.^1^H NMR (400 MHz, DMSO-*d_6_*) δ 9.04 (s, 1H), 8.71 (s, 1H), 8.10 (d, *J* = 7.1 Hz, 1H), 7.86 (s, 1H), 7.70 (d, *J* = 7.3 Hz, 1H), 7.47–7.33 (m, 2H), 4.64 (s, 1H), 4.00 (s, 3H), 3.81 (s, 2H), 3.58 (s, 2H), 2.64 (d, *J* = 22.4 Hz, 10H). ^13^C NMR (100 MHz, DMSO-*d_6_*) δ 164.41, 155.44, 155.12, 142.34, 137.22, 137.12, 125.04, 123.09, 122.12, 119.65, 111.52, 104.45, 102.90, 62.86, 59.62, 57.63, 52.95, 51.93, 33.38. HRMS: calcd for C_21_H_26_N_7_O [M + H]^+^, 392.2193, found: 392.2195.

4-((7-(1-Methyl-1*H*-indol-3-yl)-[1,2,4]triazolo[1,5-*a*]pyrimidin-5-yl)methyl)thiomorpholine 1,1-dioxide (**H17**).

White powdery solid, Yield, 57%, M.p. 151–152 °C. ^1^H NMR (400 MHz, DMSO-*d_6_*) δ 9.06 (s, 1H), 8.72 (s, 1H), 8.16 (d, *J* = 5.8 Hz, 1H), 7.84 (s, 1H), 7.70 (d, *J* = 8.0 Hz, 1H), 7.43–7.38 (m, 2H), 4.02 (s, 2H), 4.01 (s, 3H), 3.20 (s, 4H), 3.13 (s, 4H). ^13^C NMR (100 MHz, DMSO-*d_6_*) δ 163.85, 155.41, 155.19, 142.58, 137.24, 137.21, 125.06, 123.09, 122.20, 120.08, 111.40, 104.74, 102.97, 60.95, 50.55, 50.41, 33.40. HRMS: calcd for C_19_H_20_N_6_O_2_S [M + H]^+^, 397.1441, found: 397.1441.

2-(((7-(1-Methyl-1*H*-indol-3-yl)-[1,2,4]triazolo[1,5-*a*]pyrimidin-5-yl)methyl)thio)benzothiazole (**H18**).

White powdery solid, Yield, 57%, M.p. 167–168 °C. ^1^H NMR (400 MHz, DMSO-*d_6_*) δ 9.09 (s, 1H), 8.75 (s, 1H), 8.04 (d, *J* = 11.8 Hz, 3H), 7.94 (d, *J* = 8.1 Hz, 1H), 7.66 (d, *J* = 8.2 Hz, 1H), 7.51 (t, *J* = 7.6 Hz, 1H), 7.38 (dd, *J* = 18.0, 8.0 Hz, 2H), 7.17 (t, *J* = 7.5 Hz, 1H), 5.01 (s, 2H), 3.98 (s, 3H). ^13^C NMR (100 MHz, DMSO-*d_6_*) δ 165.93, 162.21, 155.41, 152.49, 142.49, 137.67, 137.18, 134.92, 126.47, 124.90, 124.67, 123.14, 122.06, 121.95, 121.21, 119.80, 111.46, 104.95, 102.69, 38.18, 33.42. HRMS: calcd for C_22_H_16_N_6_S_2_ [M + H]^+^: 429.0951, found: 429.0951.

### 4.5. Cell Culture

All the human cancer cells were purchased from were obtained from the Cell Bank of Type Culture Collection of Chinese Academy of Sciences (Shanghai, China).The MGC-803 cells (human gastric cancer cells), HCT-116 cells (human colorectal carcinoma cells) and MCF-7 cells (human breast cancer cells) used were cultured in humidified incubator at 37 °C and 5% CO_2_. The RPMI-1640 medium was supplemented with 10% fetal bovine serum, penicillin (100 U/mL) and streptomycin (0.1 mg/mL) [36].

### 4.6. MTT Assay

MGC-803 cells (human gastric cancer cells), HCT-116 cells (human colorectal carcinoma cells) and MCF-7 cells (human breast cancer cells) were seeded into 126-well plates and incubated for 24 h. Then, cells were treated with different concentrations of compounds. After another 48 h, MTT reagent (20 μL per well) was added and then incubated at 37 °C for 4 h. Formazan was then dissolved with DMSO. Absorbencies of formazan solution were measured at 4120 nm. The IC_50_ values of tested compounds were calculated by SPSS version 20.0.

### 4.7. Colony Formation Assay

5000 per well MGC-803 cells were seeded in a 6-well plate and incubated at 37 °C in 5% CO_2_ for 24 h, then treated with different concentrations of 10e. After 7 days, the culture medium was removed, the cells were washed with PBS twice, fixed with 4% paraformaldehyde and stained with 0.1% crystal violet. Cells’ images were captured with camera [37,38].

### 4.8. Western Blotting Assay

Gastric cancer cells MGC-803 were treated with different concentrations of compound **H12** (0 μM, 8 μM, 16 μM and 24 μM) for 48 h and then collected. Then the cells were lysed. The obtained protein samples were separated by SDS-PAGE electrophoresis, followed by constant current 200 mA transfer for 60 min to nitrocellulose membranes; membranes were then blocked with 5% skimmed milk for 1 h. The membranes were incubated with primary antibody overnight, washed 5 times with TBST, incubated with secondary antibody for 2 h at room temperature. Finally, membranes were subjected to luminescence and imaging using ECL.

### 4.9. Annexin V-FITC/PI Double-Staining Assay for Apoptosis Detection

MGC-803 cells were treated with different concentrations of compound **H12** (0 μM, 8 μM, 16 μM and 24 μM) for 48 h and then collected. Then, 200 μL of Annexin V solution was added to the cell and mixed. Following this, 2.5 μL of Annexin-FITC staining solution was added and incubated at 4 °C for 15 min in dark. Then, 5 μL of PI staining solution was added and incubated at 4 °C for 15 min in dark. The cells were resuspended by PBS, and the samples were analyzed by flow cytometry after filtering.

### 4.10. Cell Cycle Distribution Assay

MGC-803 cells were treated with different concentrations of compound **H12** (0 μM, 8 μM, 16 μM and 24 μM) for 48 h, then collected and fixed in 70% cold ethanol for 24 h. A total of 100 μL of RNase A solution was added to the samples, which were incubated at 37 °C for 30 min. Then, 400 μL of PI staining solution was added to the samples, mixed and incubated at 4 °C for 30 min. The samples were then analyzed by flow cytometry after filtering.

### 4.11. Real-Time Cell Analyzer (RTCA) Cell Proliferation Detection Assay

The RTCA software (Agilent Technologies Inc, Santa Clara, CA, USA) was used to set up the program and test if the culture plate was available. Then, 50 μL of medium was added to each well of the culture plate and the monitoring time set. Following this, 4000 cells per well were seeded into the culture plate, equilibrated at room temperature for 30 min and the program started. When the growth curve was stable, the drug was added to treat the cells. We stopped the program after continuing the incubation for 48 h and exported the data to obtain the growth curve.

### 4.12. Statistical Analysis

The data of three independent experiments were expressed as mean ± SD and calculated by SPSS version 20 (IBM, Almonk, New York, NY, USA).

## Figures and Tables

**Table 1:** In vitro antiproliferative activities of compounds **H1**–**H18** against three human cancer cells.

Compounds	IC_50_ (μmol/L) ^a^		
	MGC-803	HCT-116	MCF-7
**H1**	43.2 ± 0.81 ^b^	35.0 ± 0.93	45.7 ± 1.30
**H2**	49.5 ± 0.91	23.3 ± 0.80	23.3 ± 0.27
**H3**	48.5 ± 2.23	44.1 ± 1.35	68.9 ± 2.15
**H4**	58.3 ± 1.31	23.8 ± 0.61	34.7 ± 0.88
**H5**	12.4 ± 0.41	13.1 ± 0.42	28.1 ± 0.92
**H6**	59.9 ± 2.10	68.9 ± 3.87	>80
**H7**	65.0 ± 3.13	25.3 ± 0.24	64.5 ± 1.79
**H8**	>80	34.6 ± 0.87	65.4 ± 1.89
**H9**	15.1 ± 0.47	15.4 ± 0.32	>80
**H10**	35.3 ± 1.28	32.2 ± 0.52	55.9 ± 0.09
**H11**	30.5 ± 2.01	30.7 ± 2.34	52.1 ± 1.66
**H12**	9.47 ± 0.21	9.58 ± 0.48	13.1 ± 0.39
**H13**	73.9 ± 0.28	43.3 ± 0.83	74.3 ± 1.61
**H14**	>80	48.5 ± 0.54	49.8 ± 1.26
**H15**	34.1 ± 1.42	22.1 ± 2.94	31.9 ± 0.33
**H16**	72.5 ± 3.70	23.6 ± 0.56	38.8 ± 1.87
**H17**	79.3 ± 1.46	46.5 ± 1.08	62.5 ± 1.39
**H18**	17.5 ± 0.65	18.5 ± 0.51	39.1 ± 1.41
**5-Fu**	9.91 ± 0.32	18.1 ± 1.68	14.8 ± 1.02

^a^ In vitro antiproliferative activity was assayed by exposure for 48 h. ^b^ mean ± SD of three independent experiments performed in duplicate.

## Data Availability

The data presented in this study are available in supplementary material.

## Supplementary Materials

The following supporting information can be downloaded at: https://www.mdpi.com/article/10.3390/molecules27154996/s1, Figure S1: ^1^H NMR of compound **H1** (DMSO-*d_6_*, 400 MHz), Figure S2: ^13^C NMR of compound **H1** (DMSO-*d_6_*, 100 MHz), Figure S3: HR-MS of compound **H1**, Figure S4: ^1^H NMR of compound **H2** (DMSO-*d_6_*, 400 MHz), Figure S5: ^13^C NMR of compound **H2** (DMSO-*d_6_*, 400 MHz), Figure S6: HR-MS of compound **H2**, Figure S7: ^1^H NMR of compound **H3** (DMSO-*d_6_*, 400 MHz), Figure S8: ^13^C NMR of compound **H3** (DMSO-*d_6_*, 100 MHz), Figure S9: HR-MS of compound **H3**, Figure S10: ^1^H NMR of compound **H4** (DMSO-*d_6_*, 400 MHz), Figure S11: ^13^C NMR of compound **H4** (DMSO-*d_6_*, 100 MHz), Figure S12: HR-MS of compound **H4**, Figure S13: ^1^H NMR of compound **H5** (DMSO-*d_6_*, 400 MHz), Figure S14: ^13^C NMR of compound **H5** (DMSO-*d_6_*, 100 MHz), Figure S15: HR-MS of compound **H5**, Figure S16: ^1^H NMR of compound **H6** (DMSO-*d_6_*, 400 MHz), Figure S17: ^13^C NMR of compound **H6** (DMSO-*d_6_*, 100 MHz), Figure S18: HR-MS of compound **H6**, Figure S19: ^1^H NMR of compound **H7** (DMSO-*d_6_*, 400 MHz), Figure S20: ^13^C NMR of compound **H7** (DMSO-*d_6_*, 100 MHz), Figure S21: HR-MS of compound **H7**, Figure S22: ^1^H NMR of compound **H8** (DMSO-*d_6_*, 400 MHz), Figure S23: ^13^C NMR of compound **H8** (DMSO-*d_6_*, 400 MHz), Figure S24: HR-MS of compound **H8**, Figure S25: ^1^H NMR of compound **H9** (DMSO-*d_6_*, 400 MHz), Figure S26: ^13^C NMR of compound **H9** (DMSO-*d_6_*, 400 MHz), Figure S27: HR-MS of compound **H9**, Figure S28: ^1^H NMR of compound **H10** (DMSO-*d_6_*, 400 MHz), Figure S29: ^13^C NMR of compound **H10** (DMSO-*d_6_*, 100 MHz), Figure S30: HR-MS of compound **H10**, Figure S31: ^1^H NMR of compound **H11** (DMSO-d6, 400 MHz), Figure S32: ^13^C NMR of compound **H11** (DMSO-*d_6_*, 100 MHz), Figure S33: HR-MS of compound **H11**, Figure S34: ^1^H NMR of compound **H12** (DMSO-*d_6_*, 400 MHz), Figure S35: ^13^C NMR of compound **H12** (DMSO-*d_6_*, 400 MHz), Figure S36: HR-MS of compound **H12**, Figure S37: ^1^H NMR of compound **H1****3** (DMSO-*d_6_*, 400 MHz), Figure S38: ^13^C NMR of compound **H1****3** (DMSO-*d_6_*, 100 MHz), Figure S39 HR-MS of compound **H1****3**, Figure S40: ^1^H NMR of compound **H1****4** (DMSO-*d_6_*, 400 MHz), Figure S41: ^13^C NMR of compound **H1****4** (DMSO-d6, 400 MHz), Figure S42: HR-MS of compound **H1****4**. Figure S43: ^1^H NMR of compound **H15** (DMSO-*d_6_*, 400 MHz), Figure S44: ^13^C NMR of compound **H15** (DMSO-*d_6_*, 100 MHz), Figure S45: HR-MS of compound **H15**, Figure S46: ^1^H NMR of compound **H16** (DMSO-*d_6_*, 400 MHz), Figure S47: ^13^C NMR of compound **H16** (DMSO-*d_6_*, 100 MHz), Figure S48: HR-MS of compound **H16**, Figure S49: ^1^H NMR of compound **H17** (DMSO-*d_6_*, 400 MHz), Figure S50: ^13^C NMR of compound **H17** (DMSO-*d_6_*, 400 MHz), Figure S51: HR-MS of compound **H17**, Figure S52: ^1^H NMR of compound **H18** (DMSO-*d_6_*, 400 MHz), Figure S53: ^13^C NMR of compound **H1****8** (DMSO-*d_6_*, 100 MHz), Figure S54 HR-MS of compound **H1****8**.

## Sample Availability

The samples are available from the authors.
