# Supplementary material for: Design, Synthesis and Biological Evaluation of [1,2,4]Triazolo[1,5-a]pyrimidine Indole Derivatives against Gastric Cancer Cells MGC-803 via the Suppression of ERK Signaling Pathway
Source: Molecules. 2022 Aug 5;27(15):4996. doi: 10.3390/molecules27154996 (PMC9370682; doi:10.3390/molecules27154996)
Supplement: Supplementary file 1 [file molecules-27-04996-s001.zip › molecules-1803147-supplementary.pdf]

Design, Synthesis and Biological Evaluation of [1,2,4]Triazolo[1,5-*a*]pyrimidine Indole Derivatives against Gastric Cancer Cells MGC-803 via the Suppression of ERK Signaling Pathway

Guang-Xi Yu [1,†], Ying Hu [2,†], Wei-Xin Zhang [2,†], Xin-Yi Tian [3], Sai-Yang Zhang [1], , Yan Zhang [1,*], Shuo Yuan [4,*] and Jian Song [1,*]

[1] School of Basic Medical Sciences, Zhengzhou University, Zhengzhou 450001, China; ygx19990110@126.com (G.-X.Y.); saiyangz@zzu.edu.cn (S.-Y.Z.)
[2] Guana'anmen Hospital China Academy of Chinese Medicinal Sciences, Beijing 100053, China; huyingdr@126.com (Y.H.); zhangweixin@gs.zzu.edu.cn (W.-X.Z.)
[3] Key Laboratory of Advanced Drug Preparation Technologies (Ministry of Education) , Institute of Drug Discovery & Development, School of Pharmaceutical Sciences , Zhengzhou University, Zhengzhou 450001, China; txyi626@163.com
[4] Children's Hospital Affiliated of Zhengzhou University, Henan Children's Hospital, Zhengzhou Children's Hospital, Zhengzhou 450018, China
***** Correspondence: zhangyan055@zzu.edu.cn (Y.Z.); 15890141701@163.com (S.Y.); mumuandzz@163.com (J.S.)
† These authors contributed equally to this work.

- ^{1}H NMR of Compound **H1**

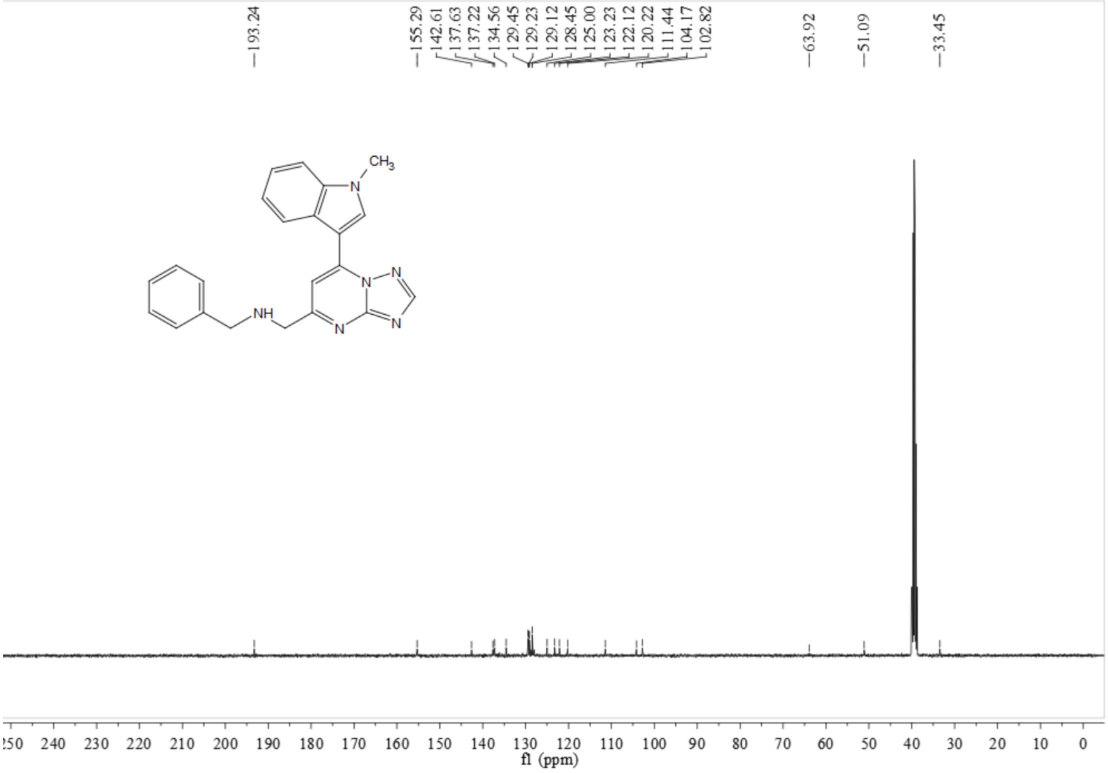

Figure S1. ^{1}H NMR spectrum of compound **H1** (400 MHz, DMSO-*d6*)

- ^{13}C NMR of Compound **H1**

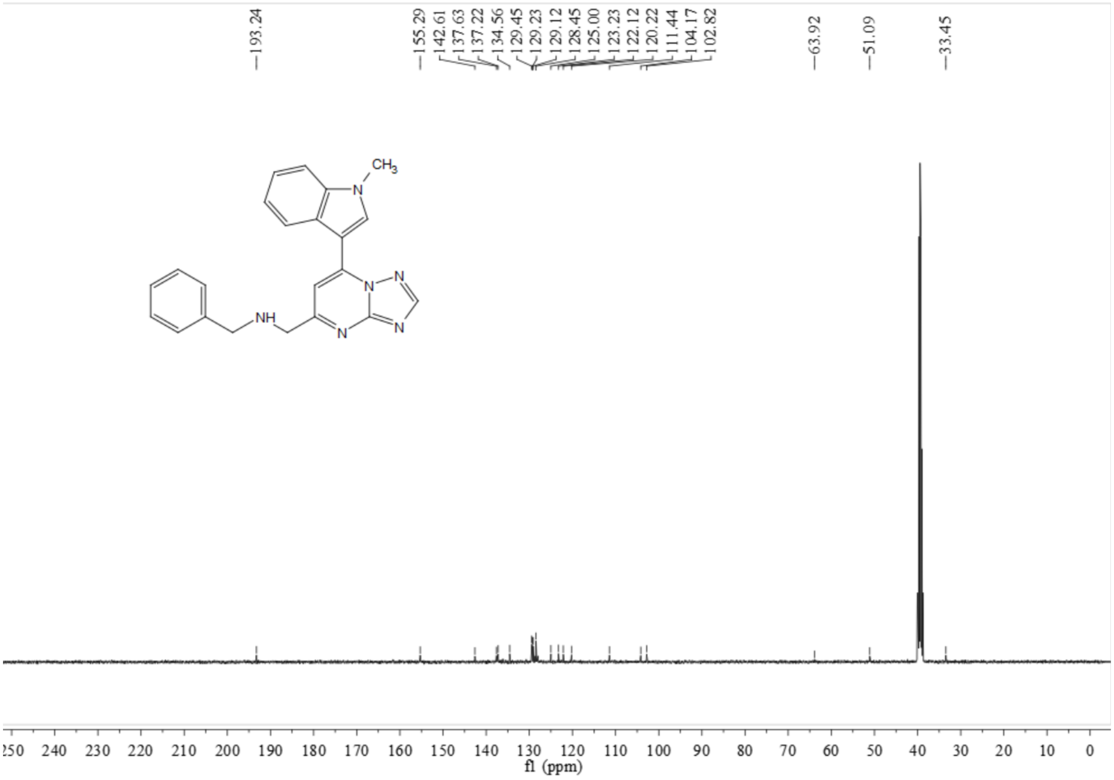

Figure S2. ^{13}C NMR spectrum of compound **H1** (100 MHz, DMSO-*d6*)

- HRMS of Compound **H1**

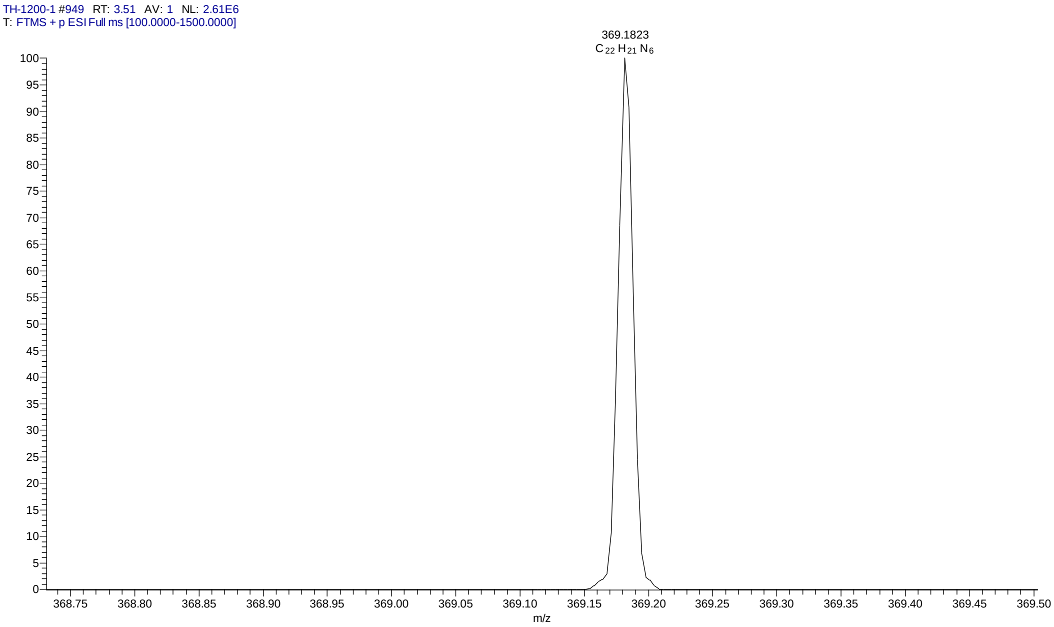

Figure S3. HRMS spectrum of compound **H1**

- ^{1}H NMR of Compound **H2**

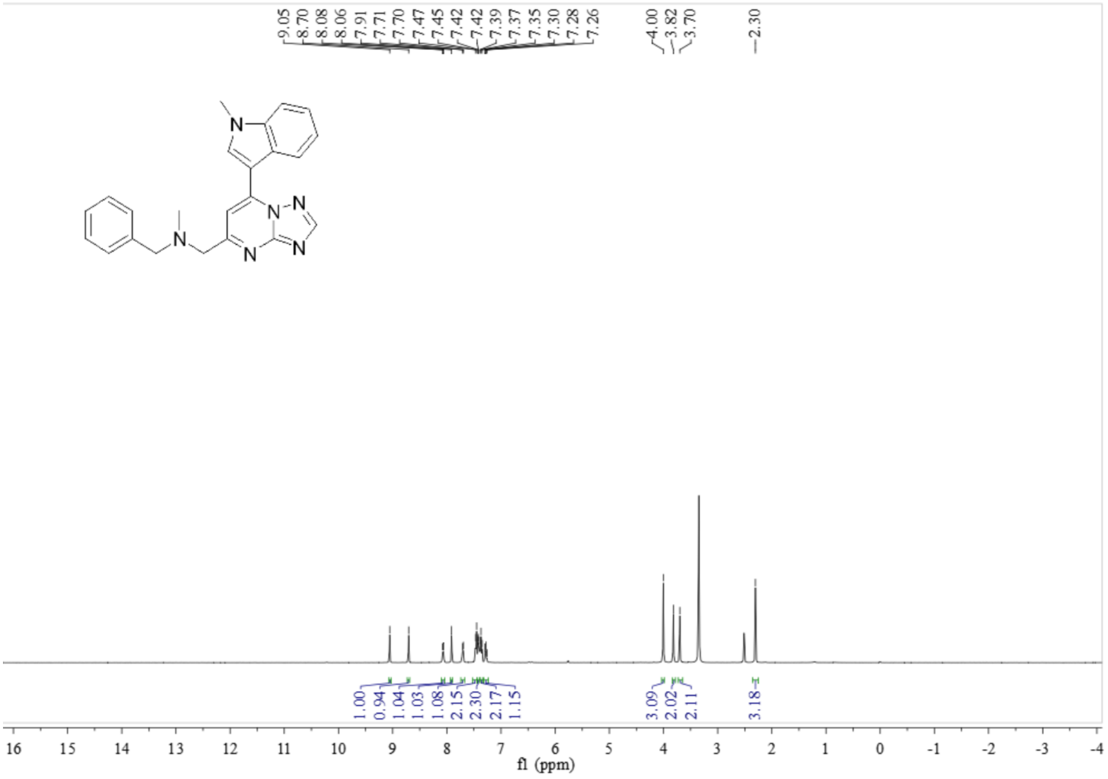

Figure S4. ^{13}C NMR spectrum of compound **H2** (100 MHz, DMSO-*d6*)

- ^{13}C NMR of Compound **H2**

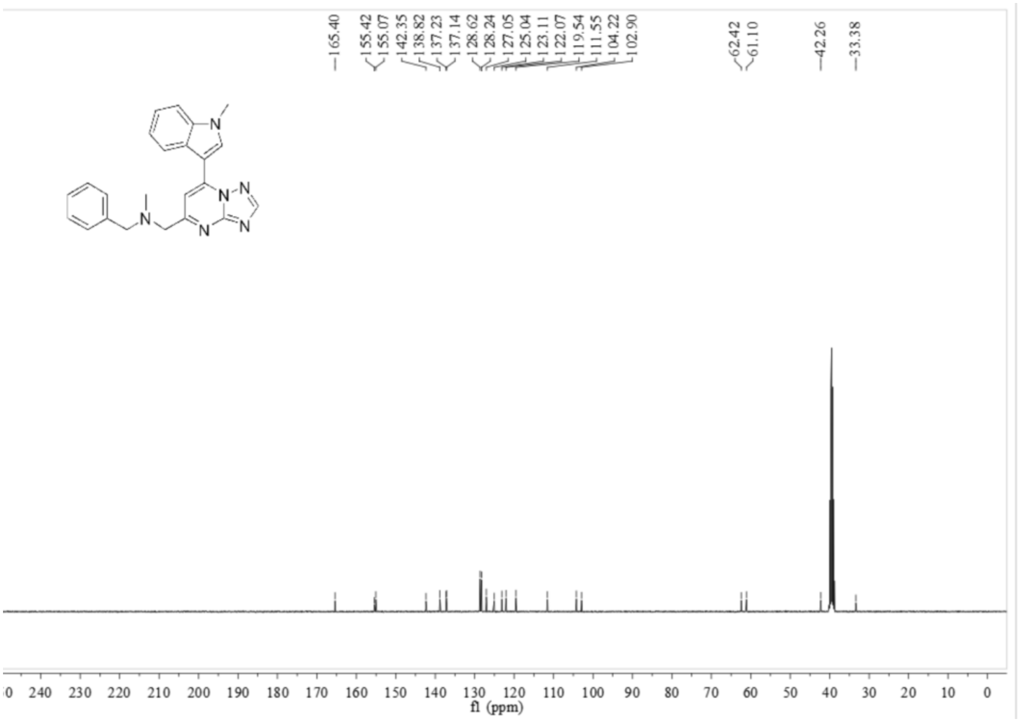

Figure S5. ^{13}C NMR spectrum of compound **H2** (100 MHz, DMSO-*d6*)

- HRMS of Compound **H2**

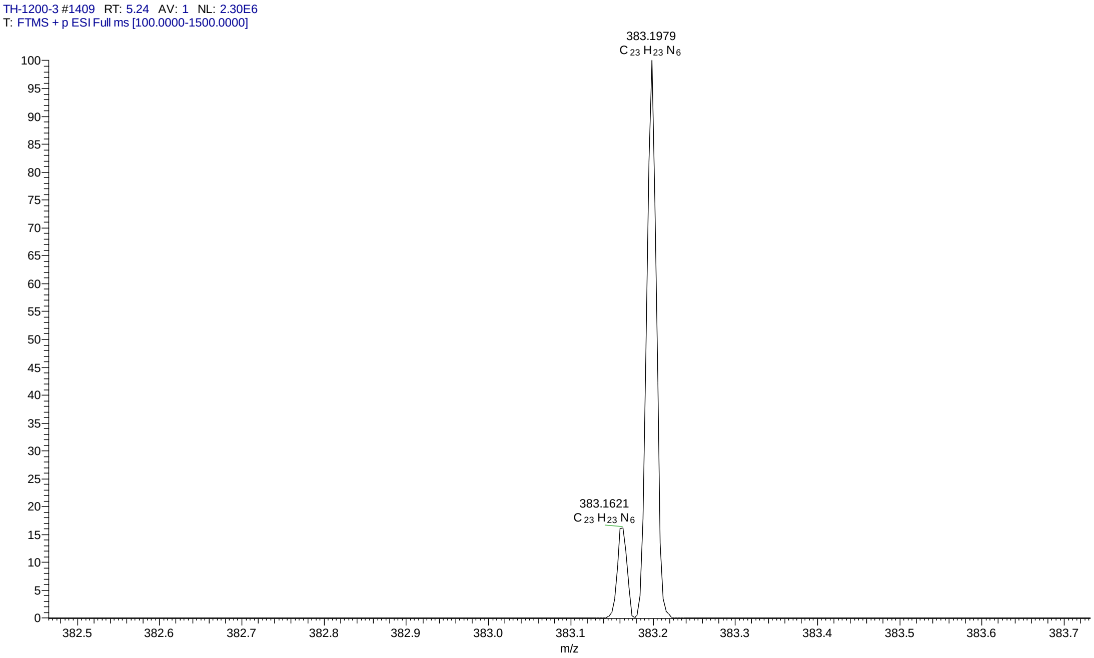

Figure S6. HRMS spectrum of compound **H2**

- ^{1}H NMR of Compound **H3**

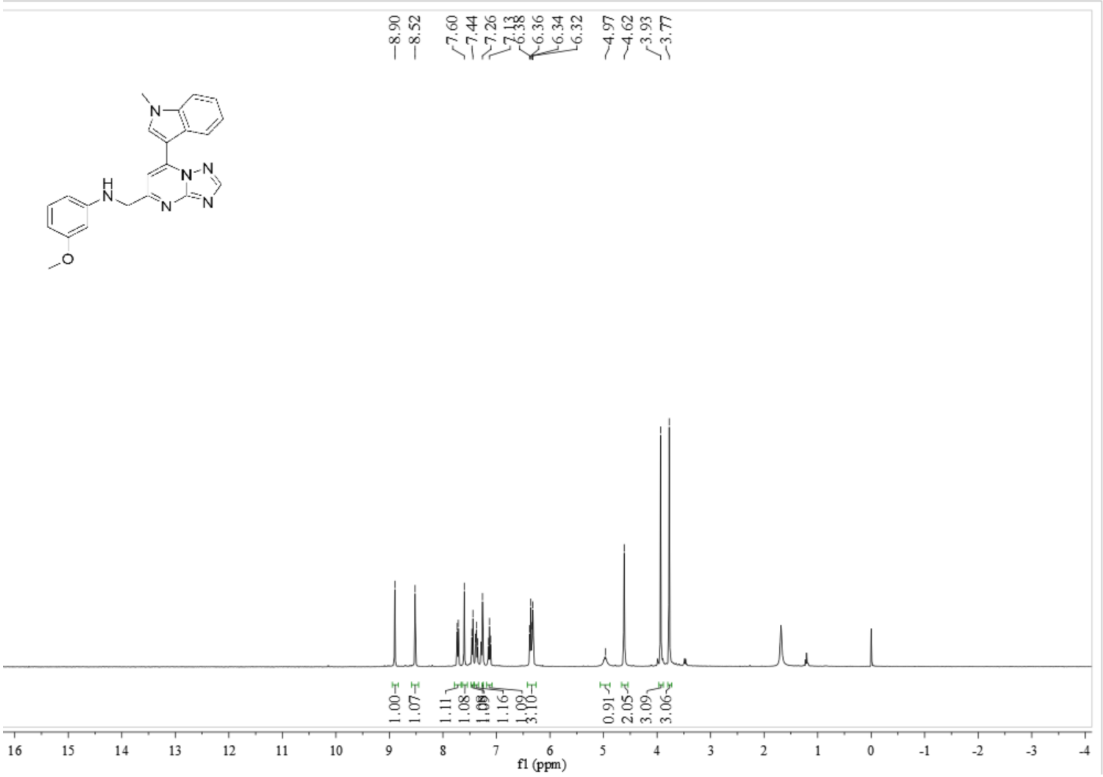

Figure S7. ^{13}C NMR spectrum of compound **H3** (100 MHz, $CDCl_3$)

- ^{13}C NMR of Compound **H3**

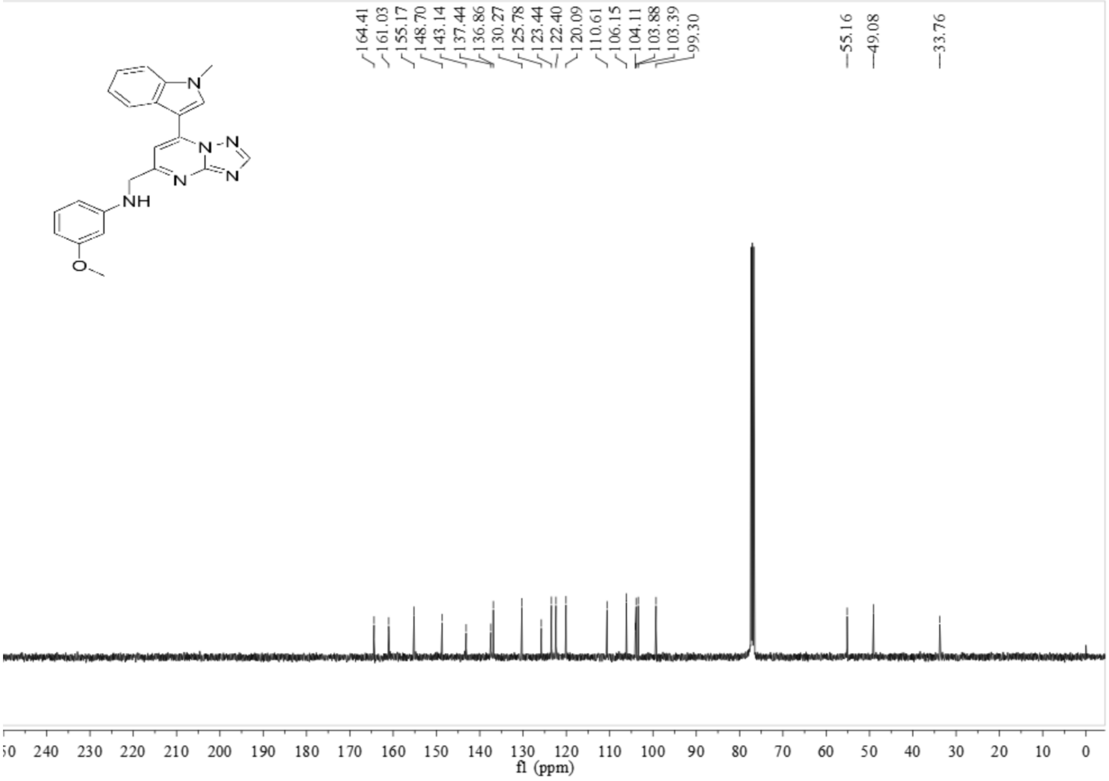

Figure S8. ^{1}H NMR spectrum of compound **H3** (400 MHz, $CDCl_3$)

- HRMS of Compound **H3**

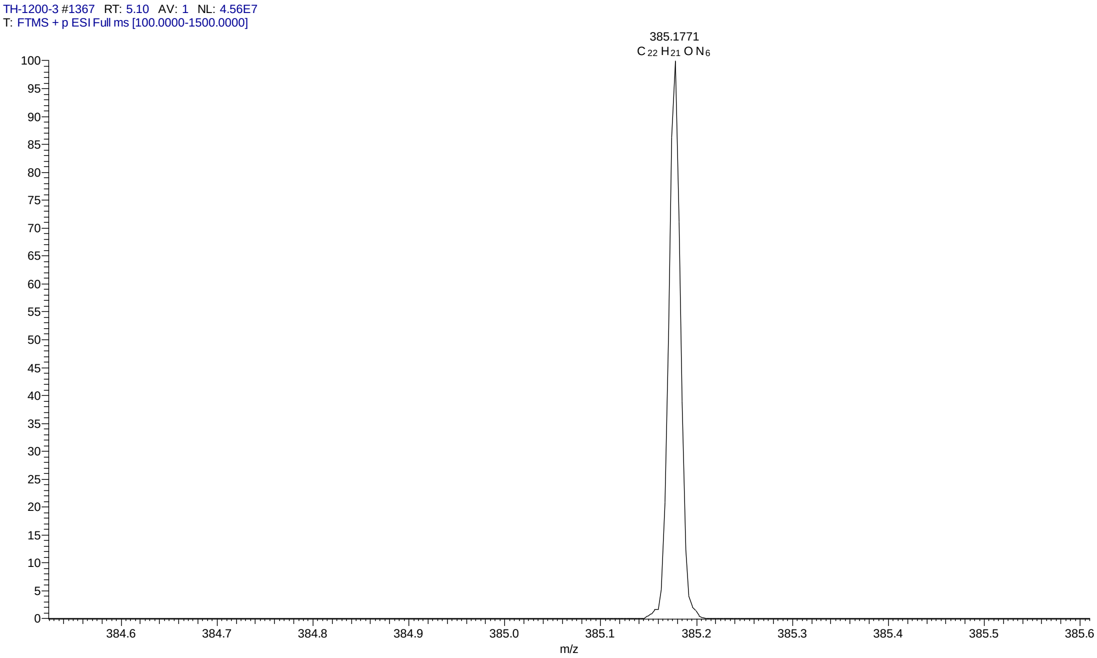

Figure S9. HRMS spectrum of compound **H3**

- ^{1}H NMR of Compound **H4**

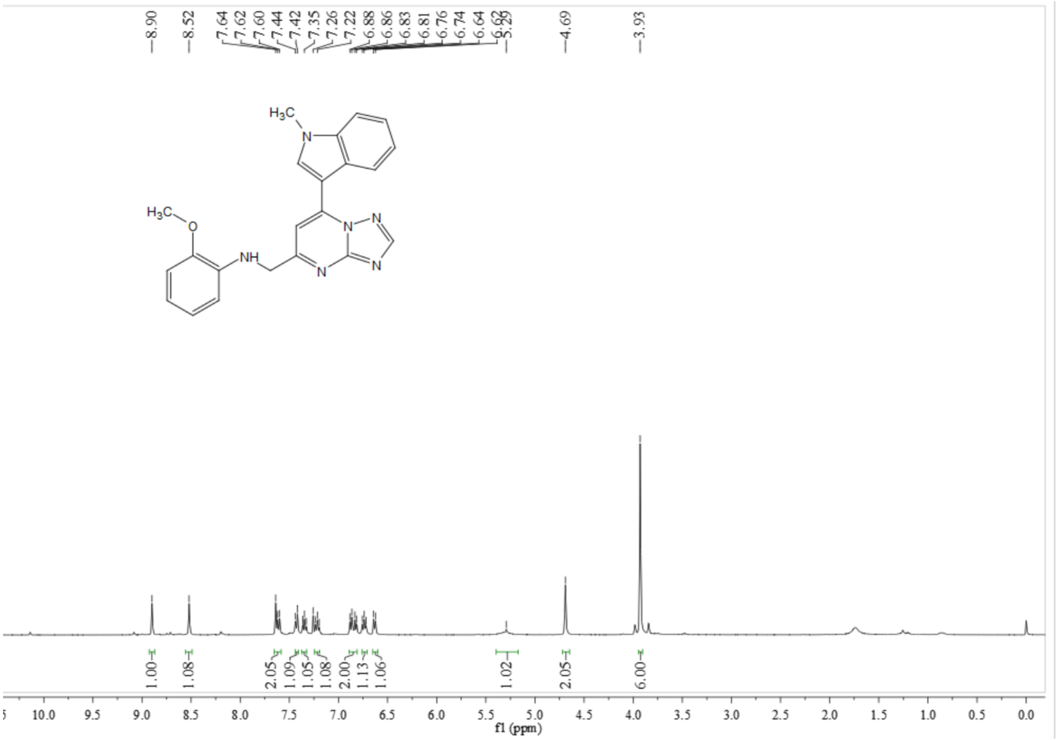

Figure S10. ^{1}H NMR spectrum of compound **H4** (400 MHz, $CDCl_3$)

- ^{13}C NMR of Compound **H4**

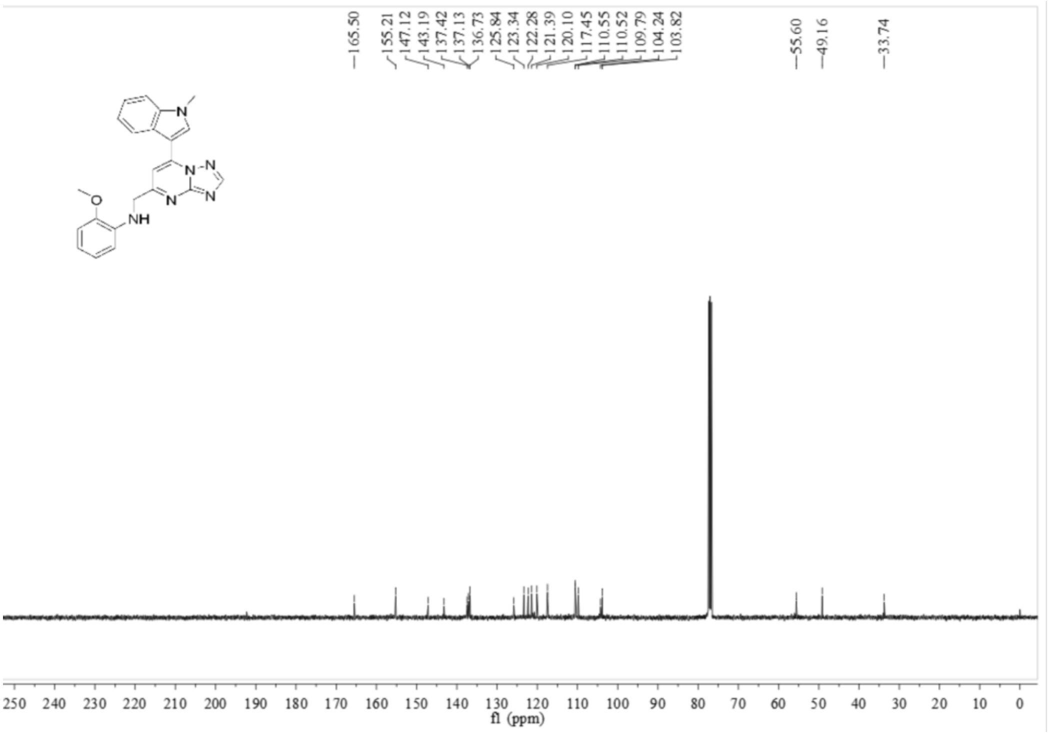

Figure S11. ^{13}C NMR spectrum of compound **H4** (100 MHz, $CDCl_3$)

- HRMS of Compound **H4**

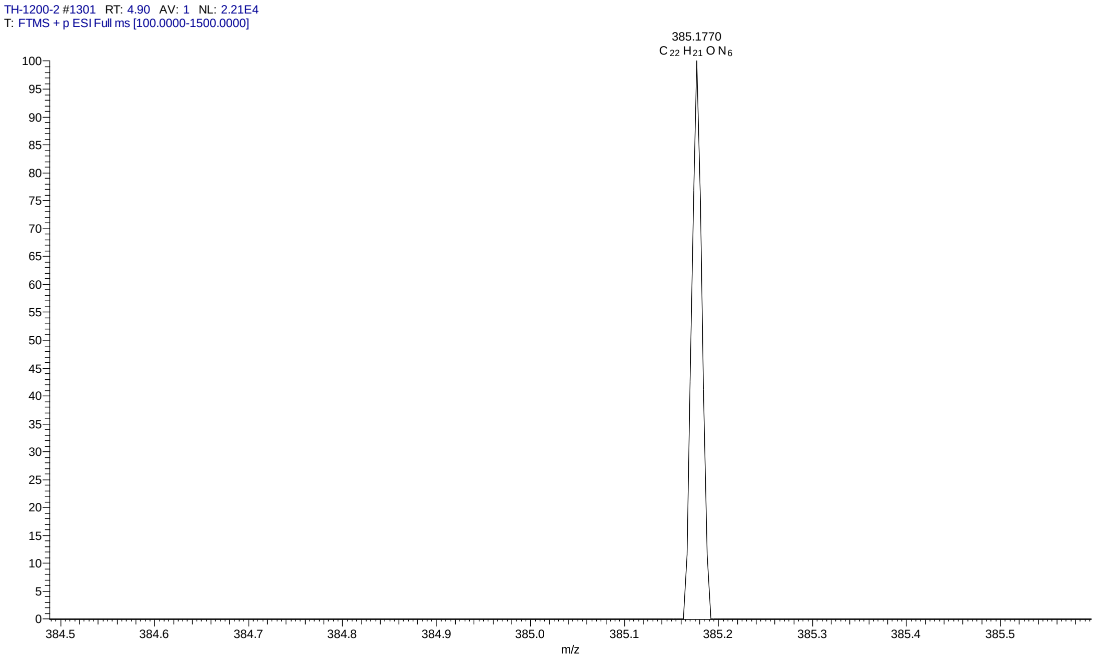

Figure S12. HRMS spectrum of compound **H4**

- ^{1}H NMR of Compound **H5**

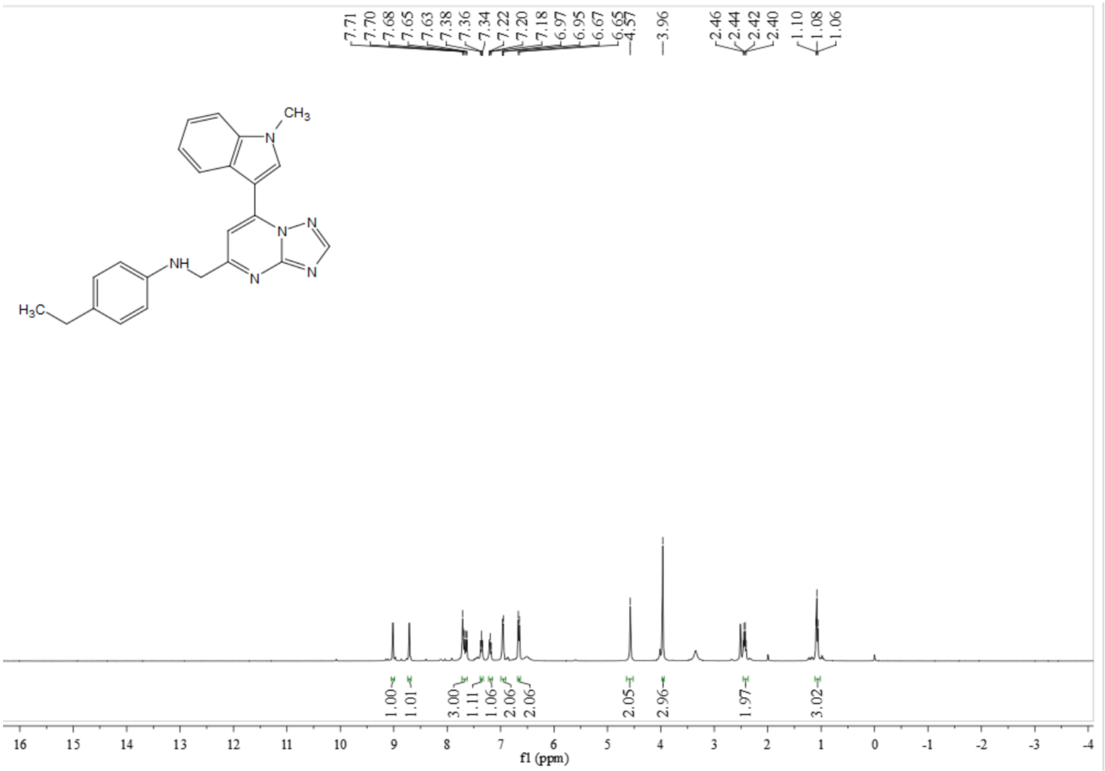

- **Figure S13**. ^{13}C NMR spectrum of compound **H5** (400 MHz, DMSO-*d6*)

- ^{13}C-NMR of Compound **H5**

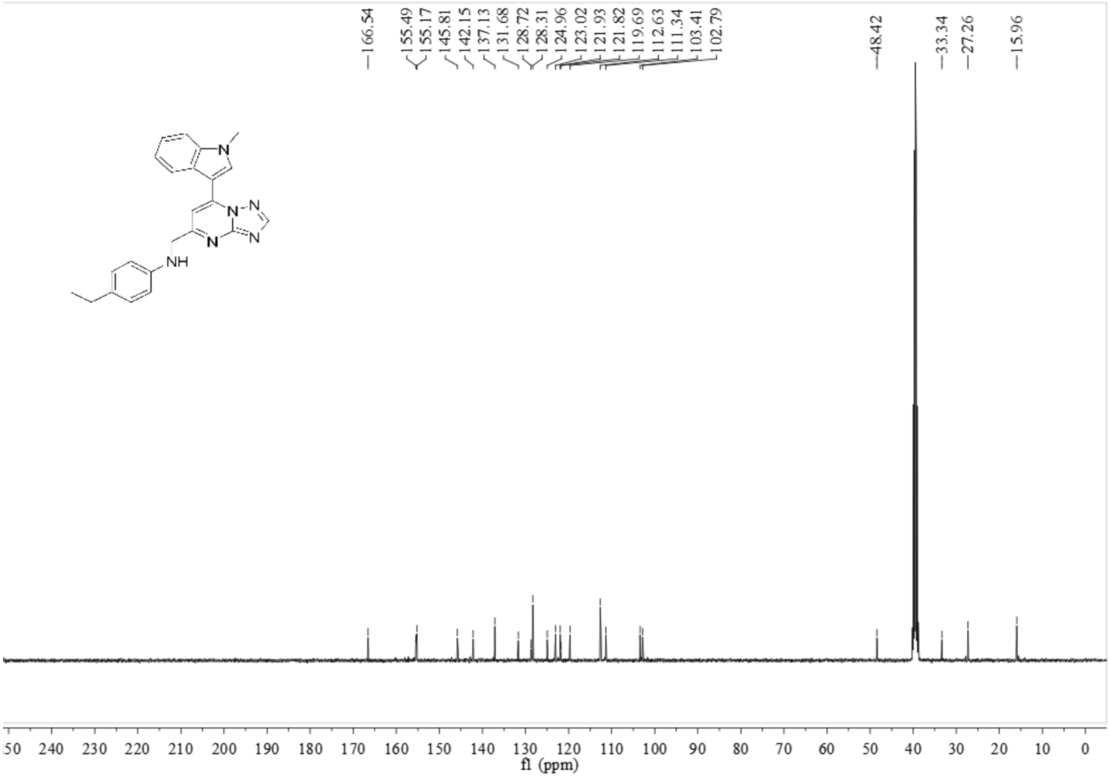

Figure S14. ^{13}C NMR spectrum of compound **H5** (100 MHz, DMSO-*d6*)

- HRMS of Compound **H5**

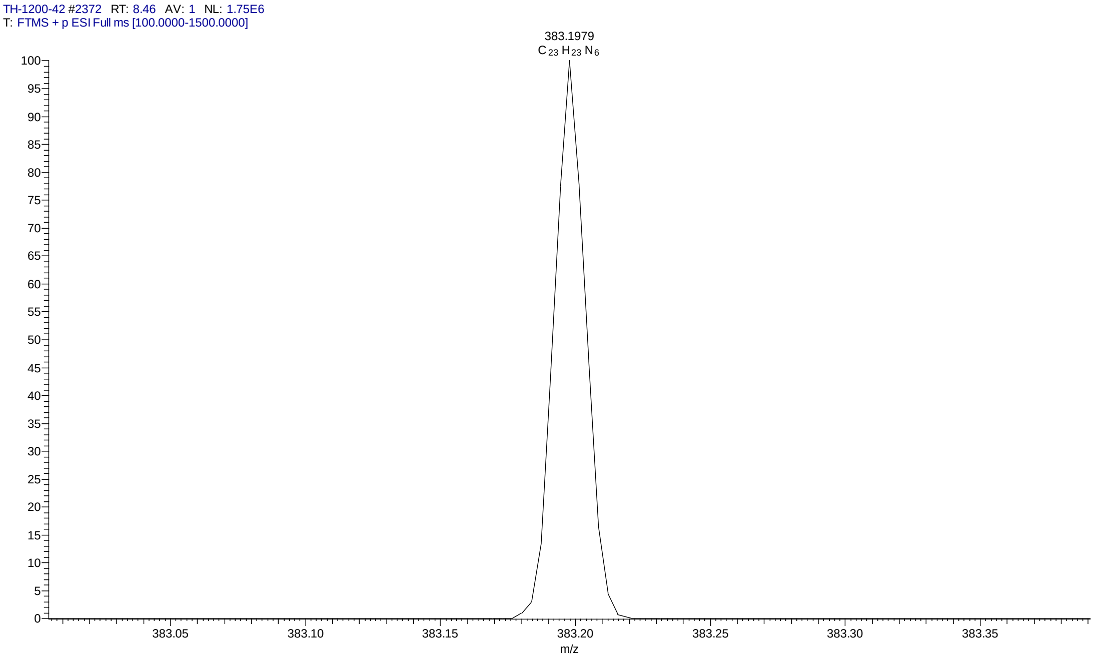

Figure S15. HRMS spectrum of compound **H5**

- ^{1}H NMR of Compound **H6**

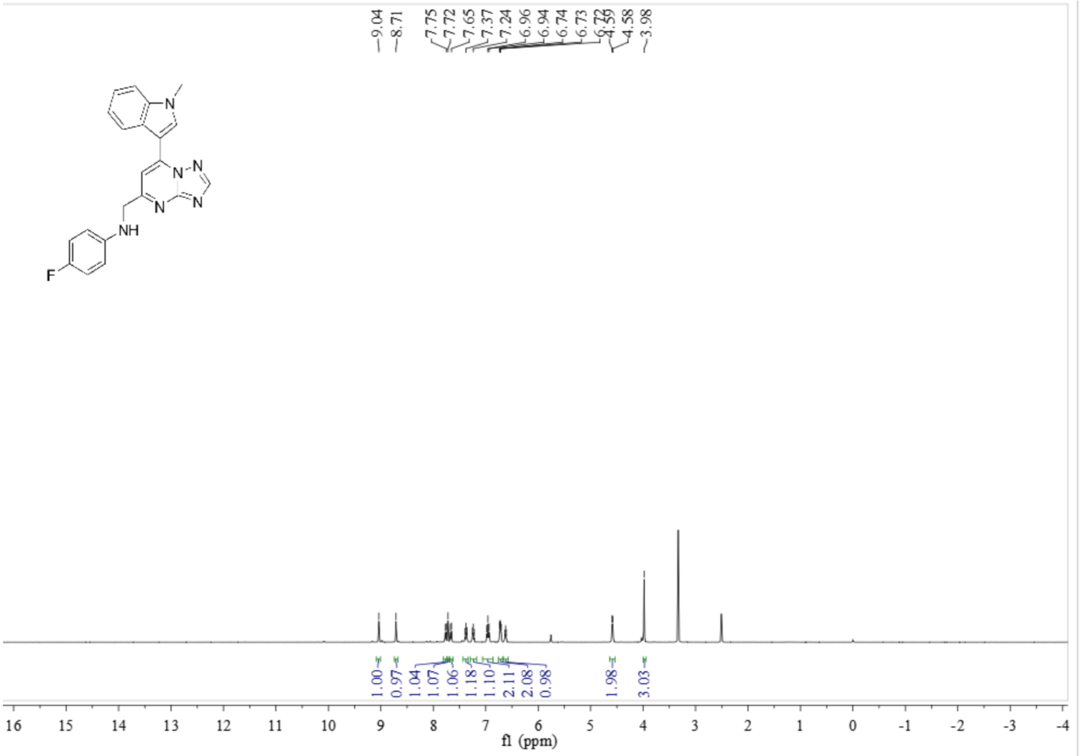

Figure S16. ^{1}H NMR spectrum of compound **H6** (400 MHz, DMSO-*d6*)

- ^{13}C-NMR of Compound **H6**

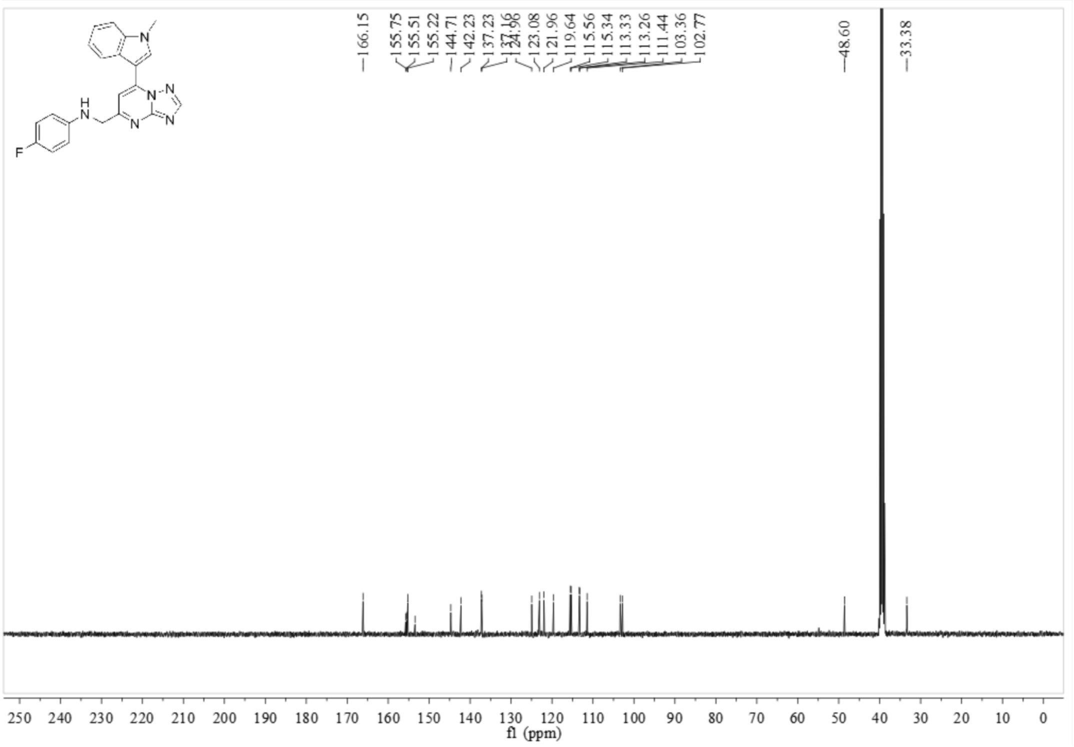

Figure S17. ^{13}C NMR spectrum of compound **H6** (100 MHz, DMSO-*d6*)

- HRMS of Compound **H6**

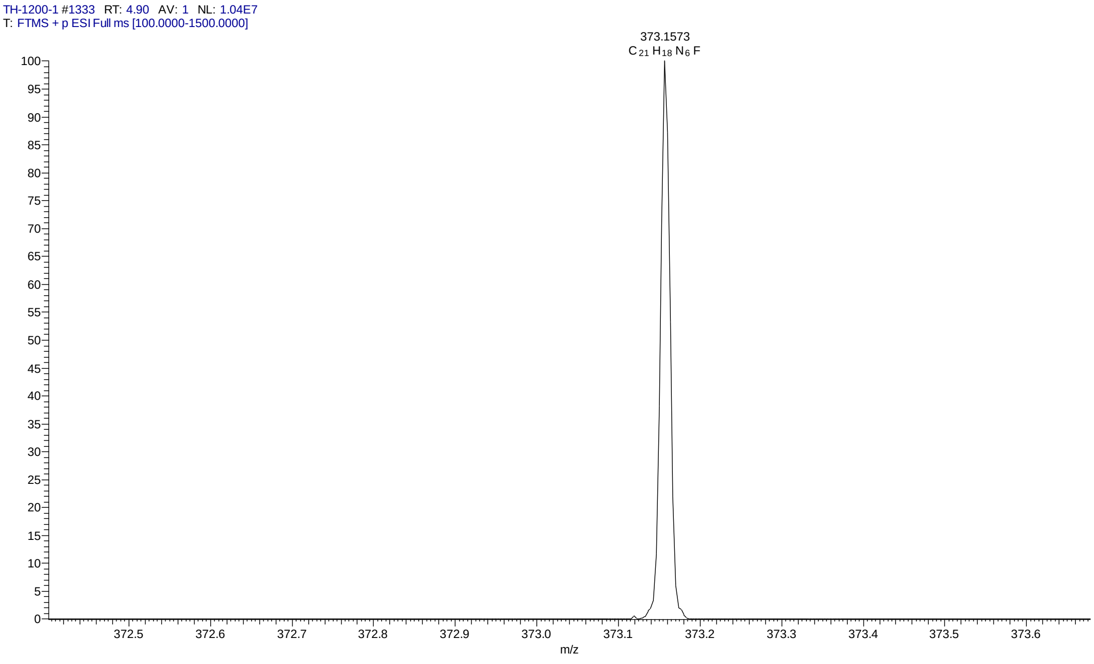

Figure S18. HRMS spectrum of compound **H6**

- ^{1}H NMR of Compound **H7**

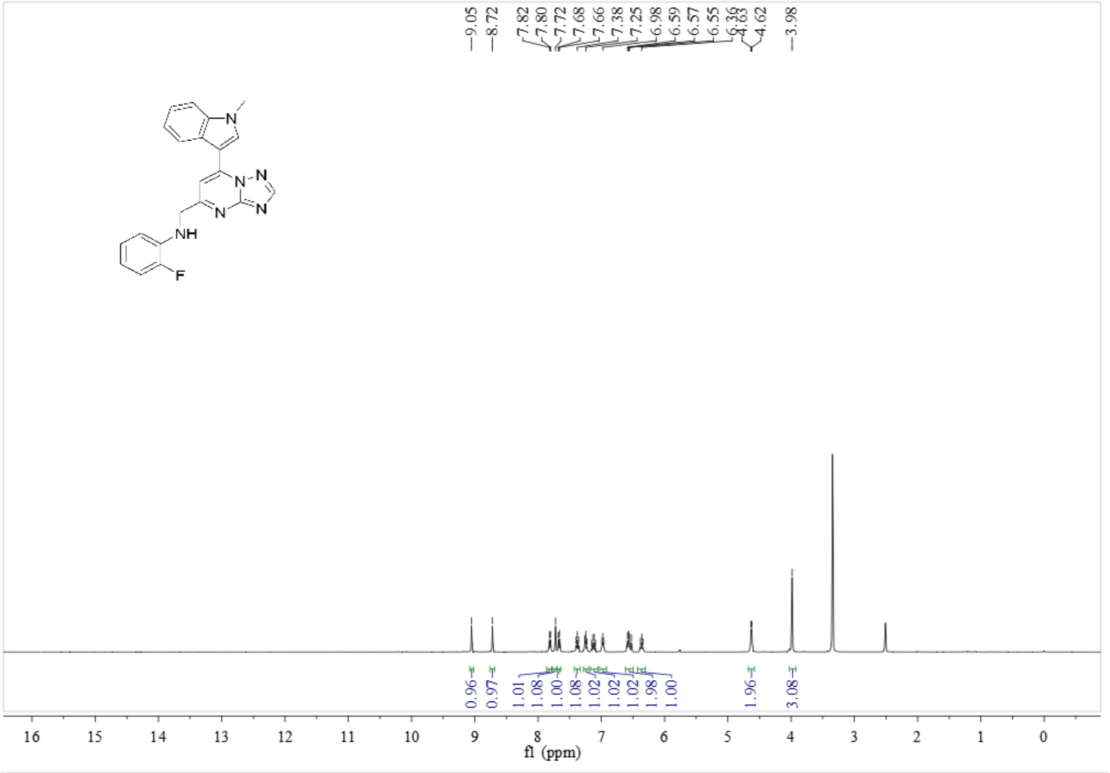

Figure S19. ^{1}H NMR spectrum of compound **H7** (400 MHz, DMSO-*d6*)

- ^{13}C-NMR of Compound **H7**

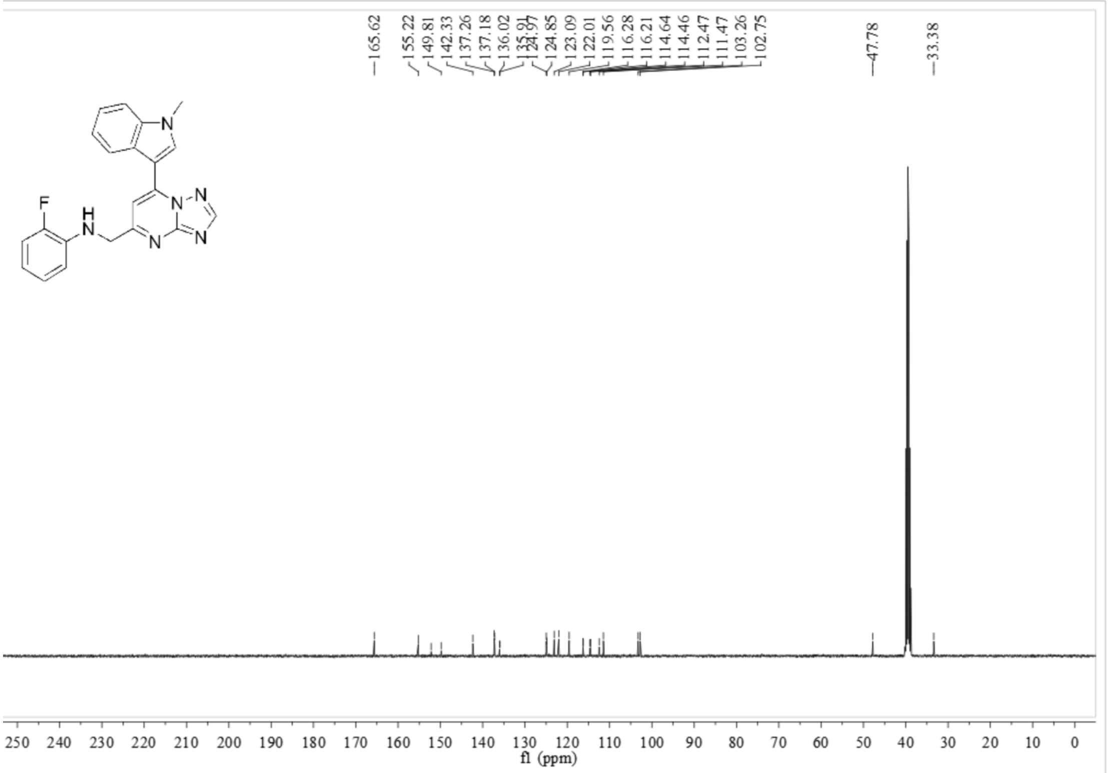

Figure S20. ^{13}C NMR spectrum of compound **H7** (100 MHz, DMSO-*d6*)

- HRMS of Compound **H7**

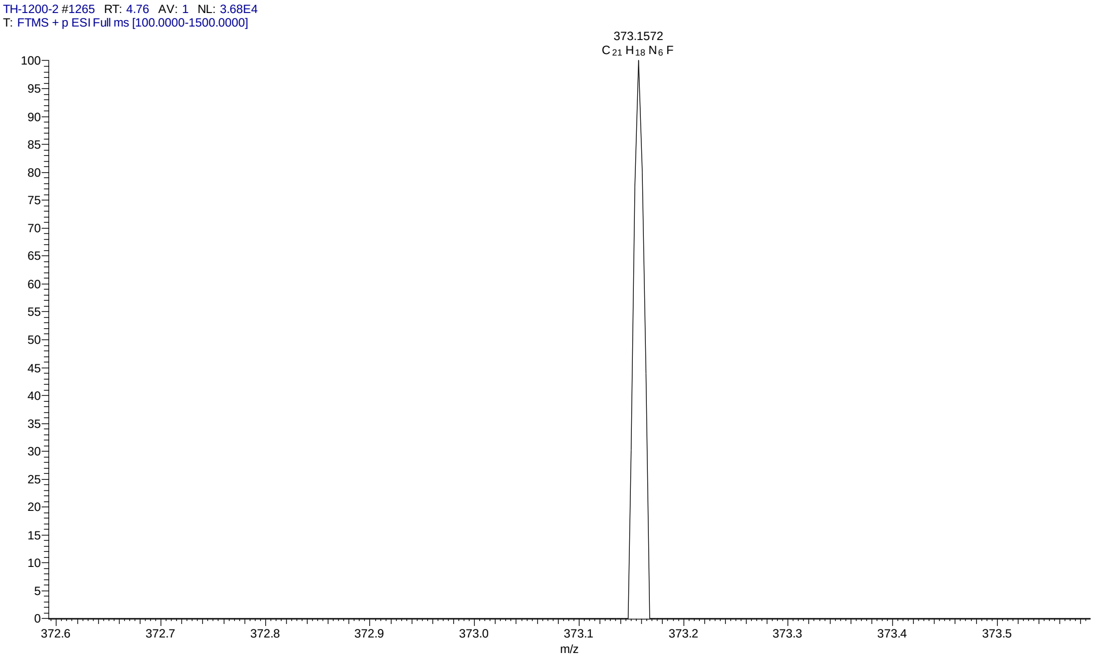

Figure S21. HRMS spectrum of compound **H7**

- ^{1}H NMR of Compound **H8**

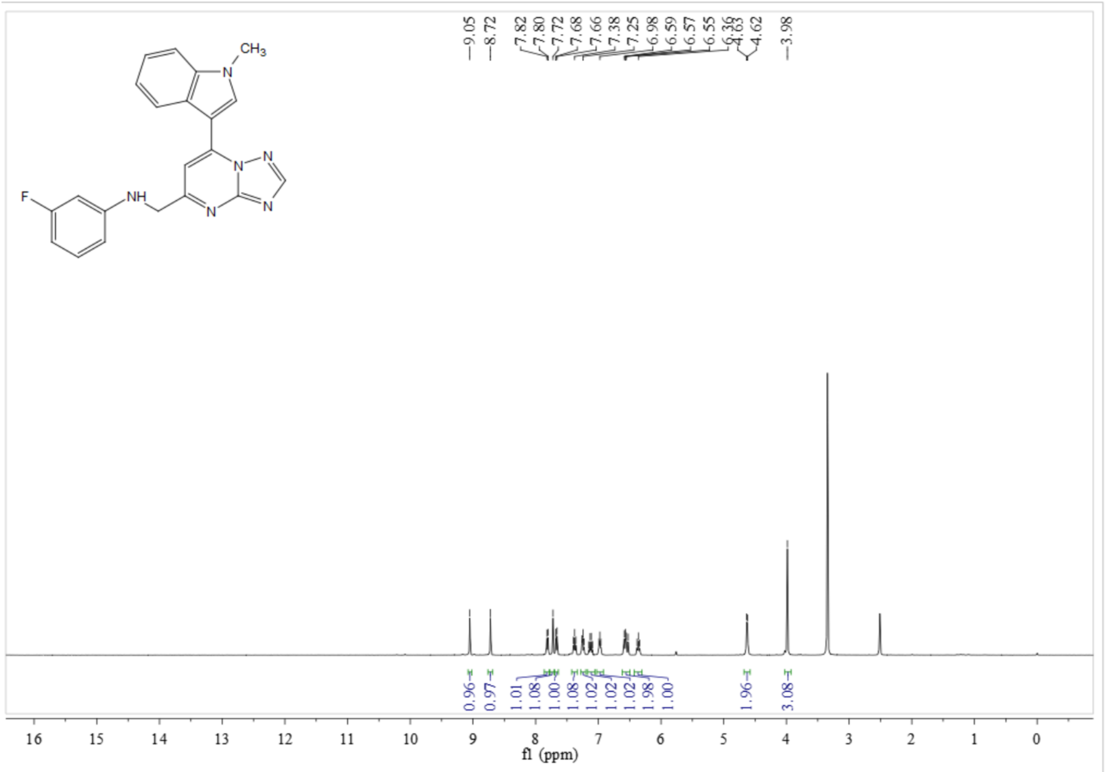

Figure S22. ^{1}H NMR spectrum of compound **H8** (400 MHz, DMSO-*d6*)

- ^{13}C-NMR of Compound **H8**

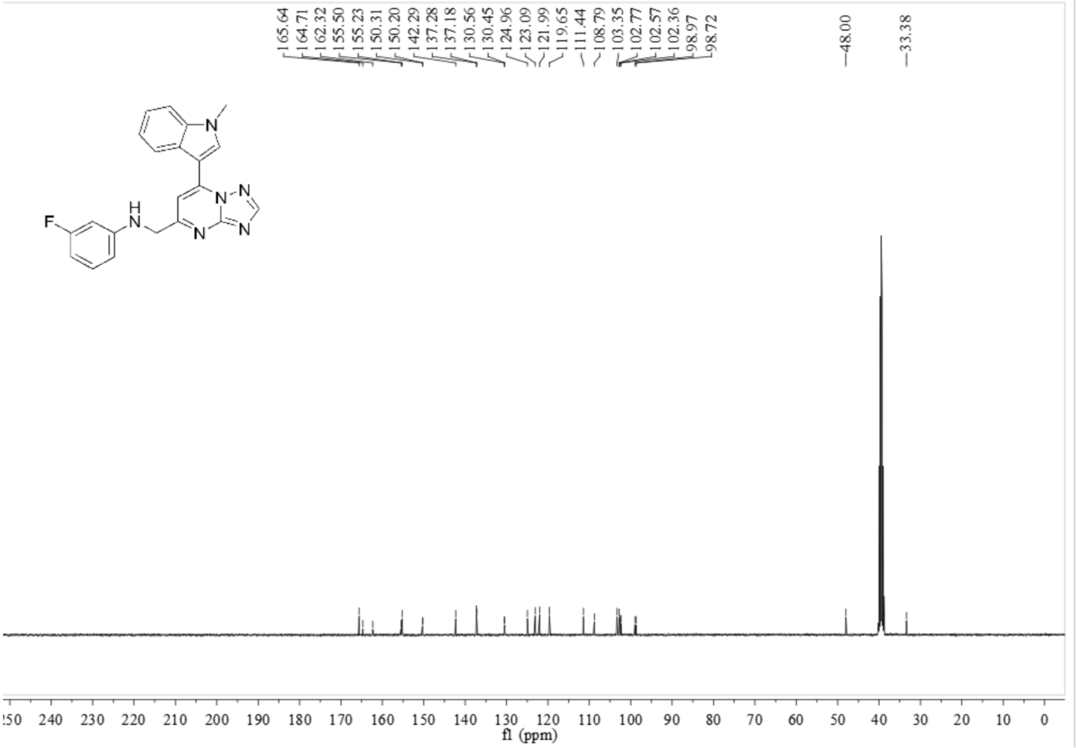

Figure S23. ^{13}C NMR spectrum of compound **H8** (100 MHz, DMSO-*d6*)

- HRMS of Compound **H8**

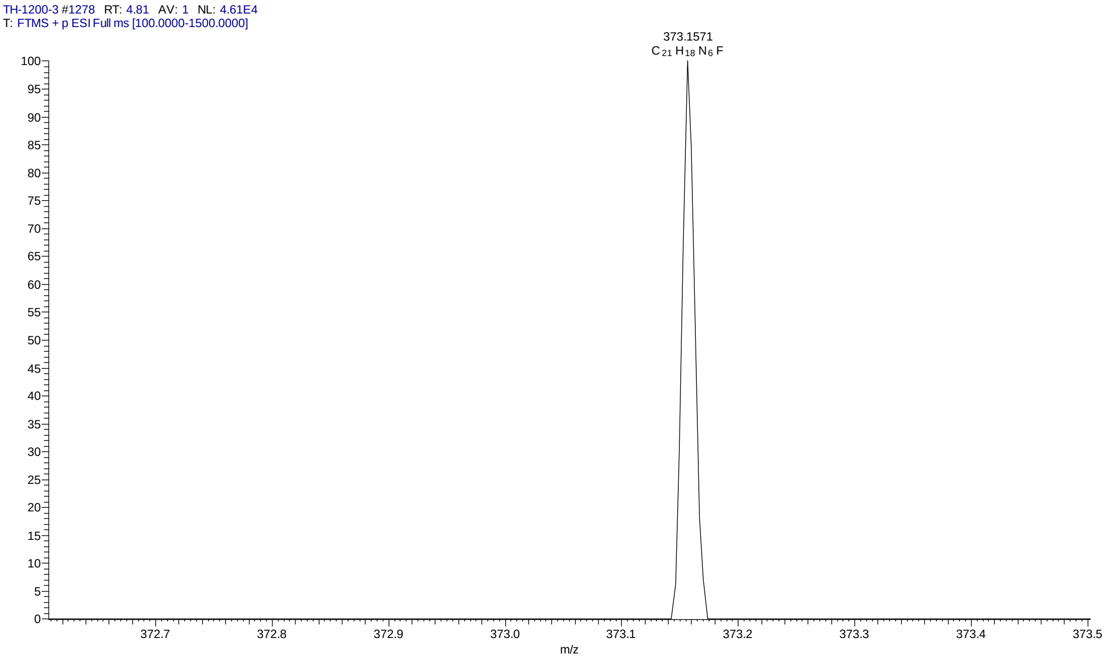

Figure S24. HRMS spectrum of compound **H8**

- ^1H NMR of Compound **H9**

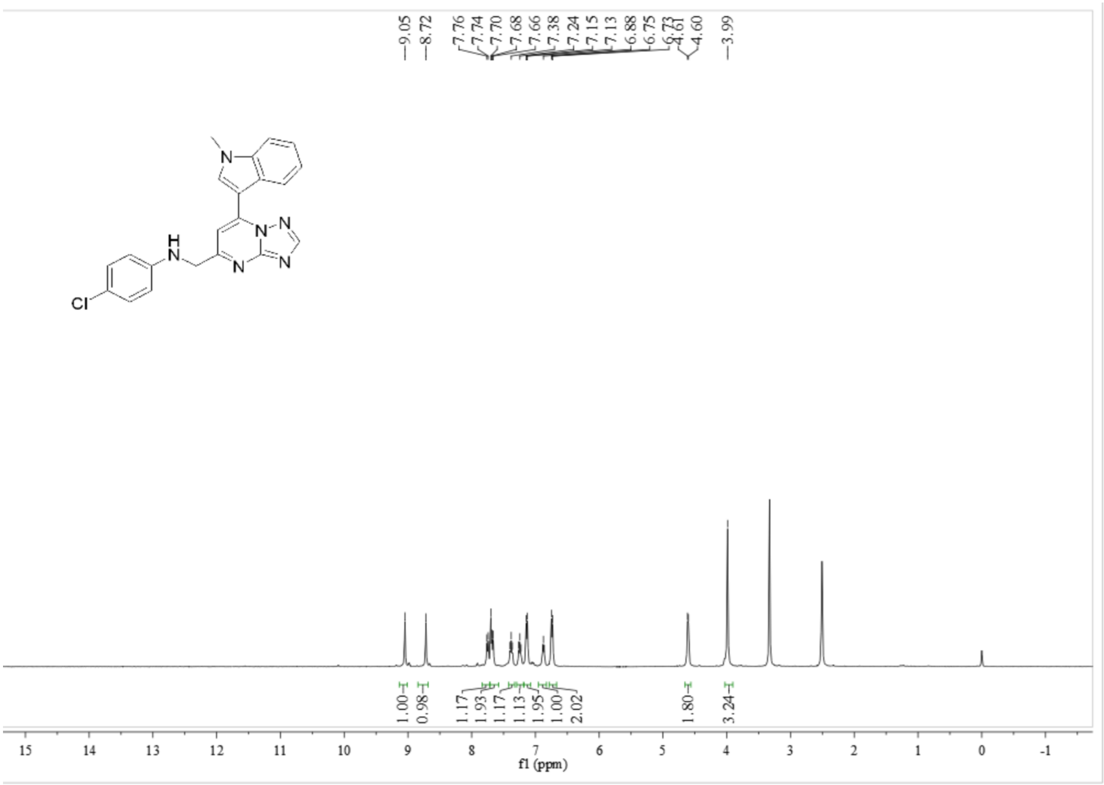

Figure S25. ^1H NMR spectrum of compound **H9** (400 MHz, DMSO-*d6*)

- ^{13}C-NMR of Compound **H9**

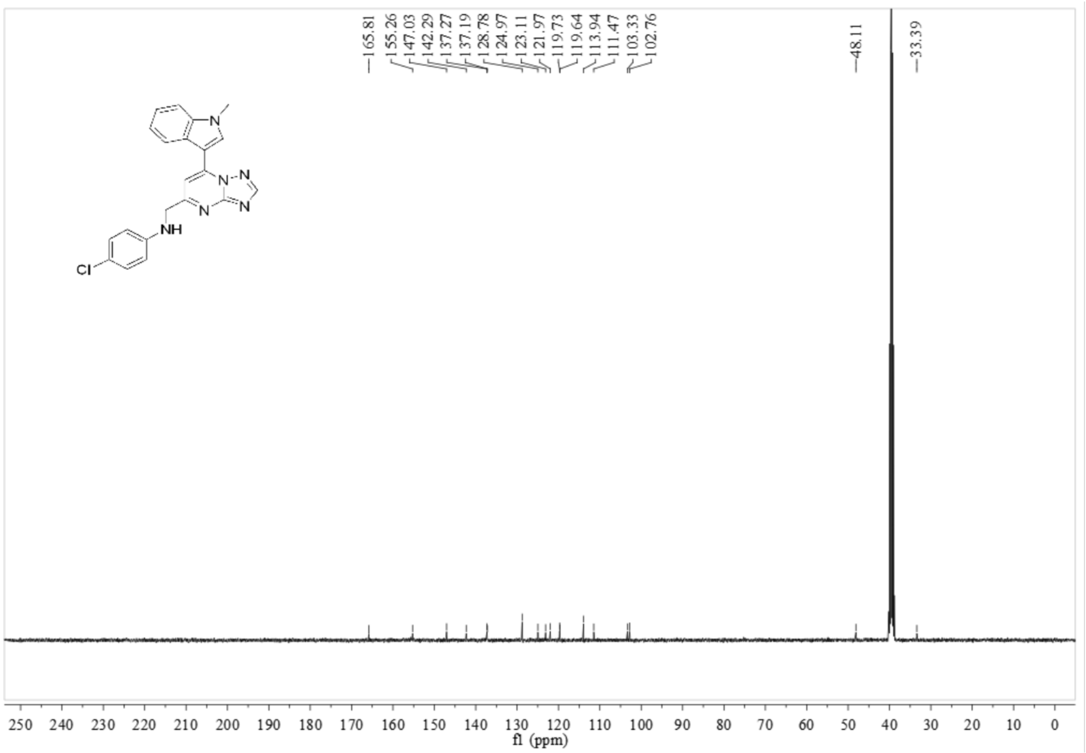

Figure S26. ^{13}C NMR spectrum of compound **H9** (100 MHz, DMSO-*d6*)

- HRMS of Compound **H9**

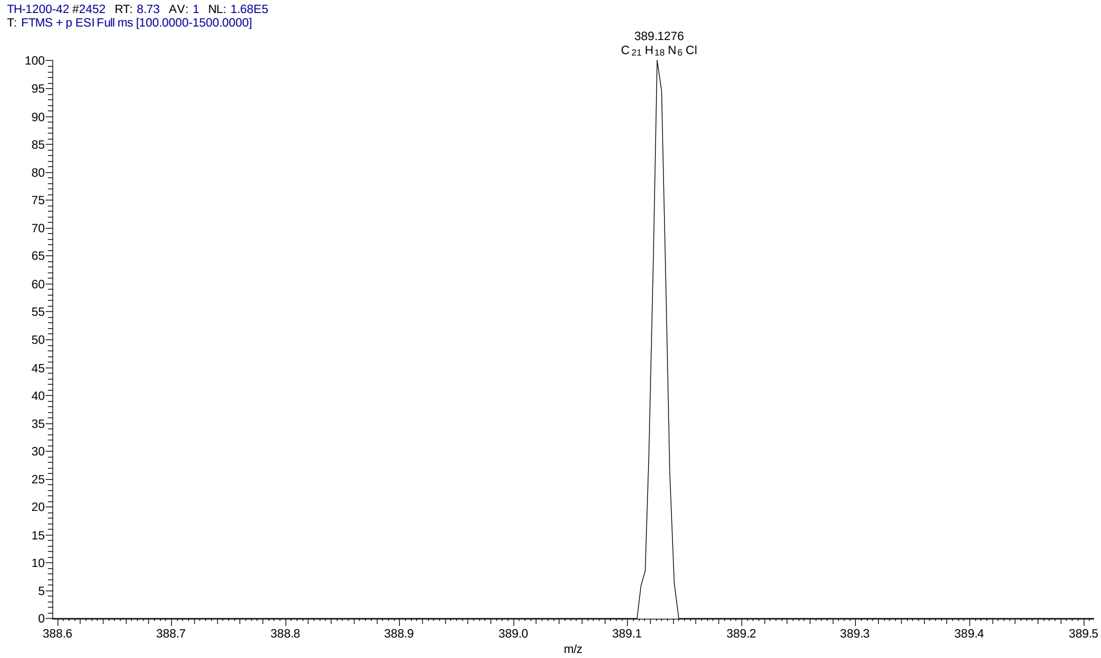

Figure S27. HRMS spectrum of compound **H9**

- ^{1}H NMR of Compound **H10**

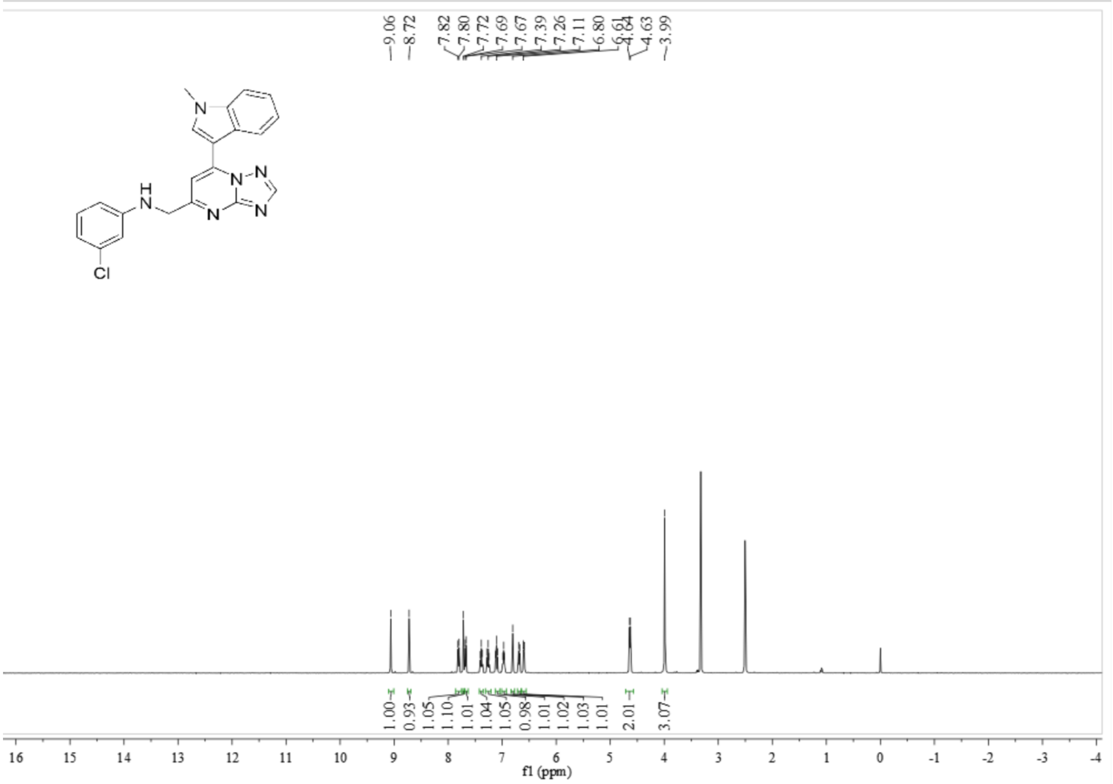

、**Figure S28**. ^{1}H NMR spectrum of compound **H10** (400 MHz, DMSO-*d6*)

- ^{13}C-NMR of Compound **H10**

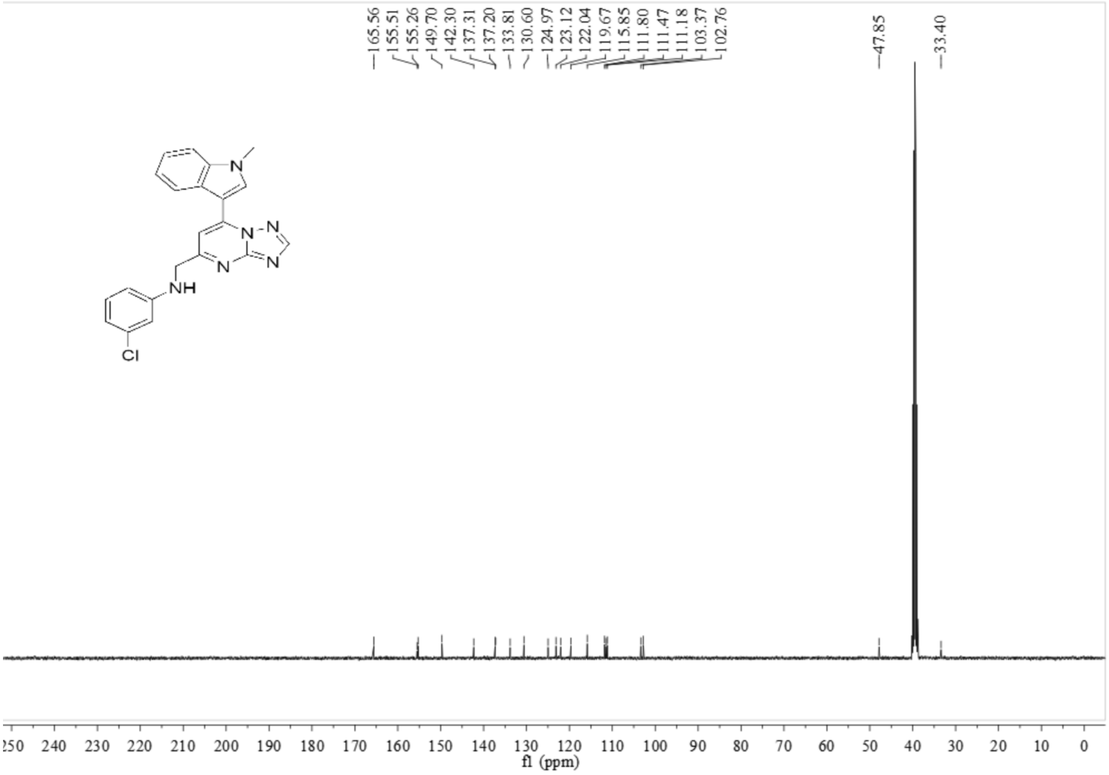

Figure S29. ^{13}C NMR spectrum of compound **H10** (100 MHz, DMSO-*d6*)

- HRMS of Compound **H10**

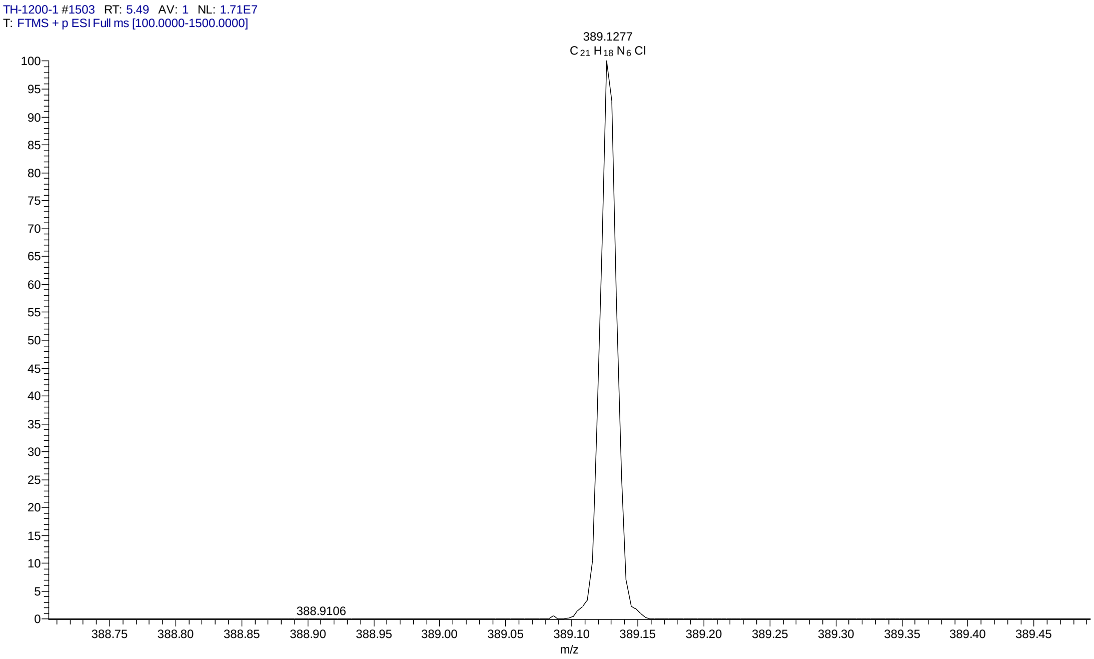

Figure S30. HRMS spectrum of compound **H10**

● ^{1}H NMR of Compound **H11**

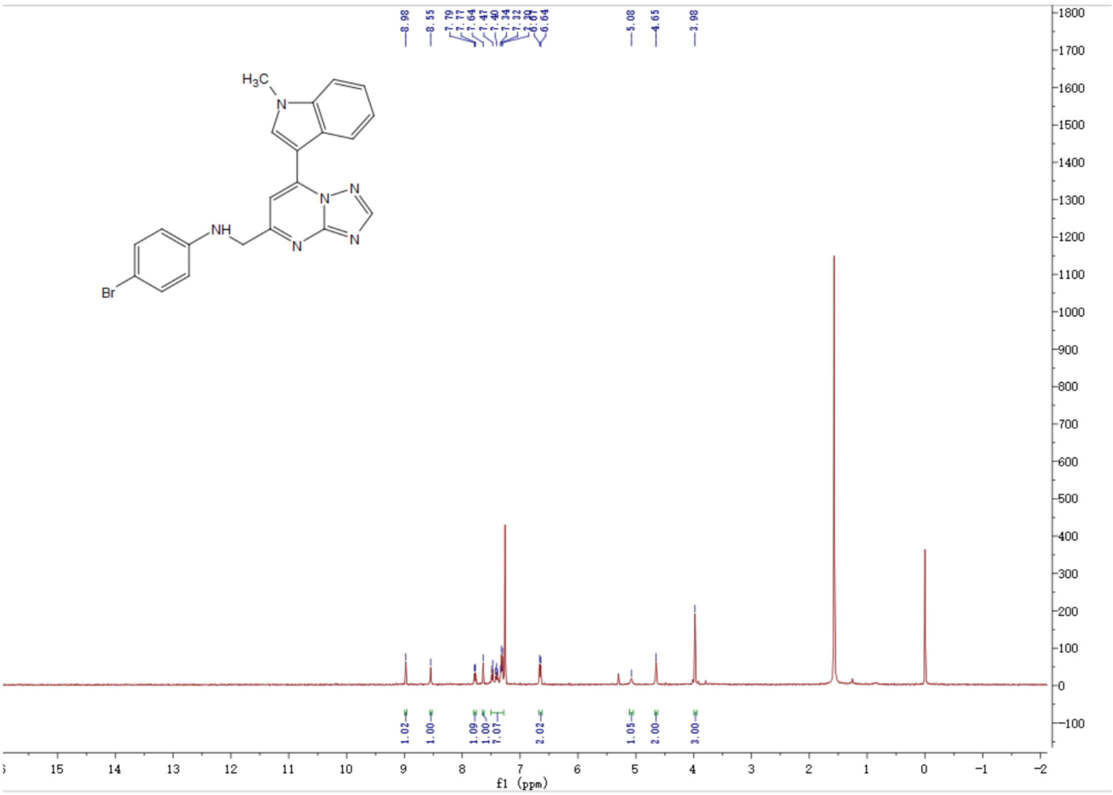

Figure S31. ^{1}H NMR spectrum of compound **H11** (400 MHz, DMSO-$CDCl_3$)

● ^{13}C-NMR of Compound **H11**

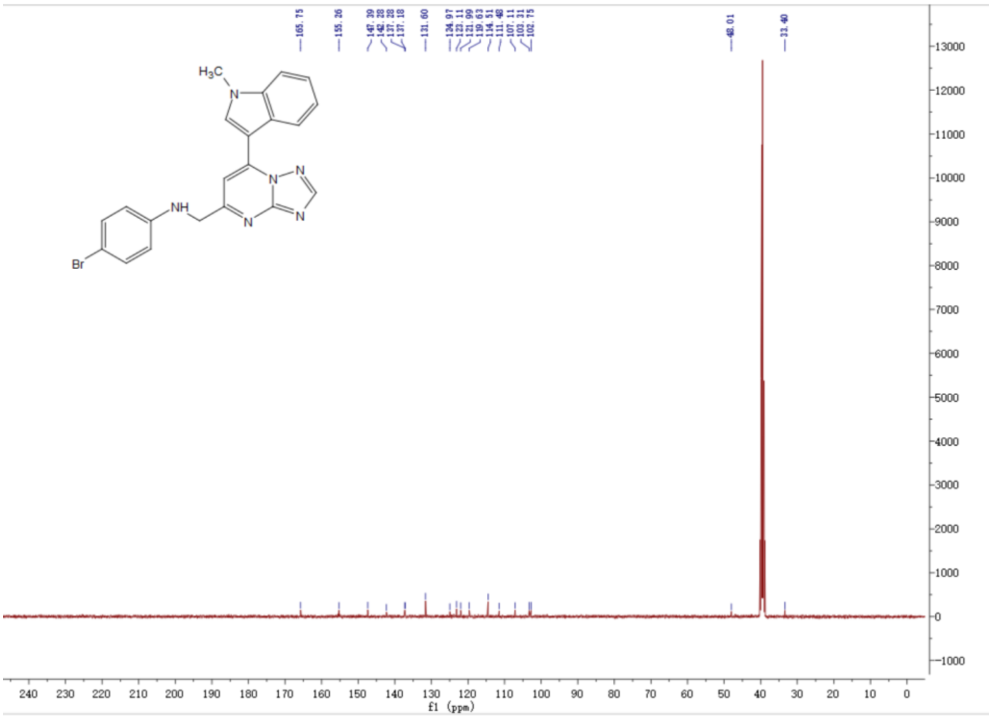

Figure S32. ^{13}C NMR spectrum of compound **H11** (100 MHz, DMSO-*d6*)

- HRMS of Compound **H11**

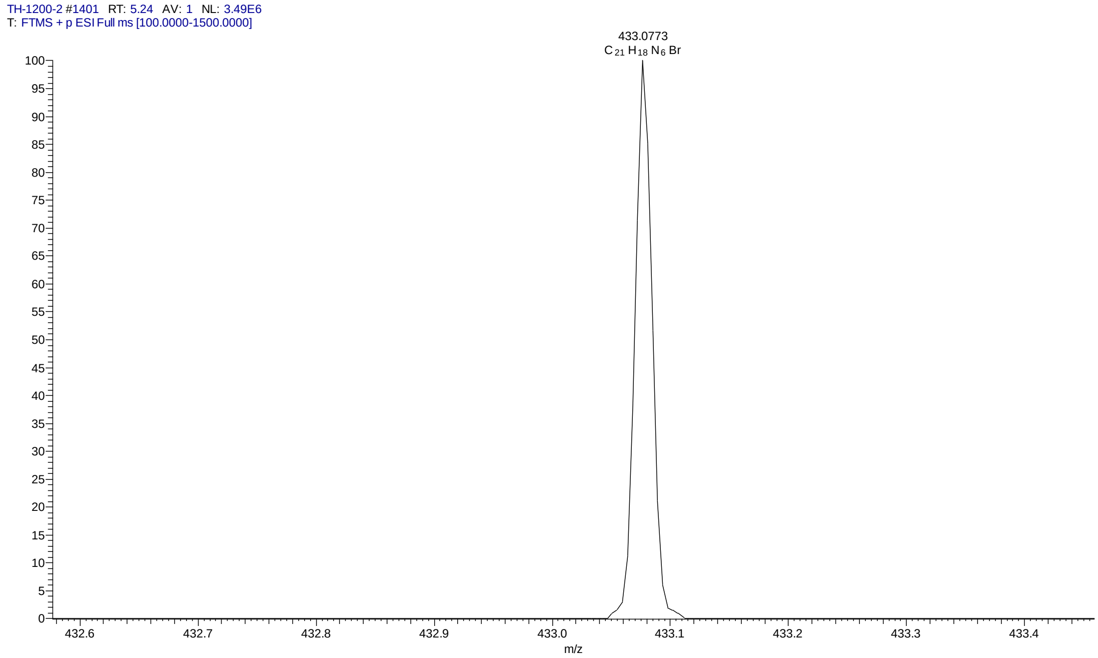

Figure S33. HRMS spectrum of compound **H11**

- ^{1}H NMR of Compound **H12**

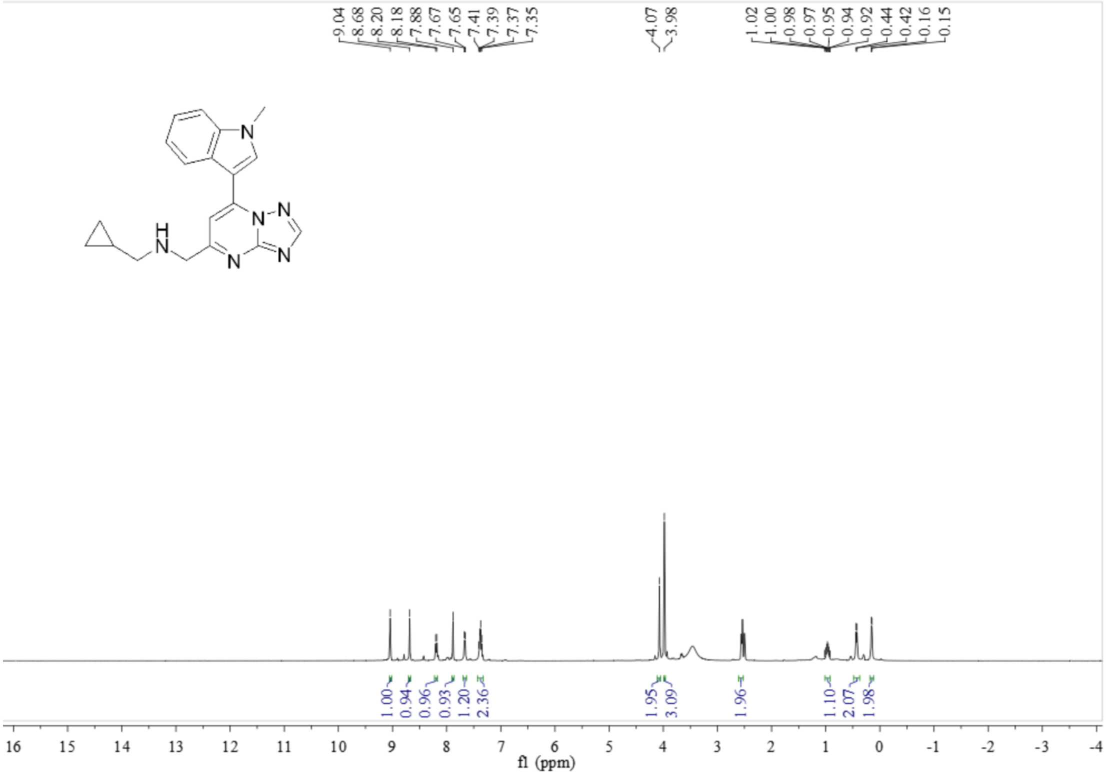

Figure S34. ^{1}H NMR spectrum of compound **H12** (400 MHz, DMSO-*d6*)

- ^{13}C-NMR of Compound **H12**

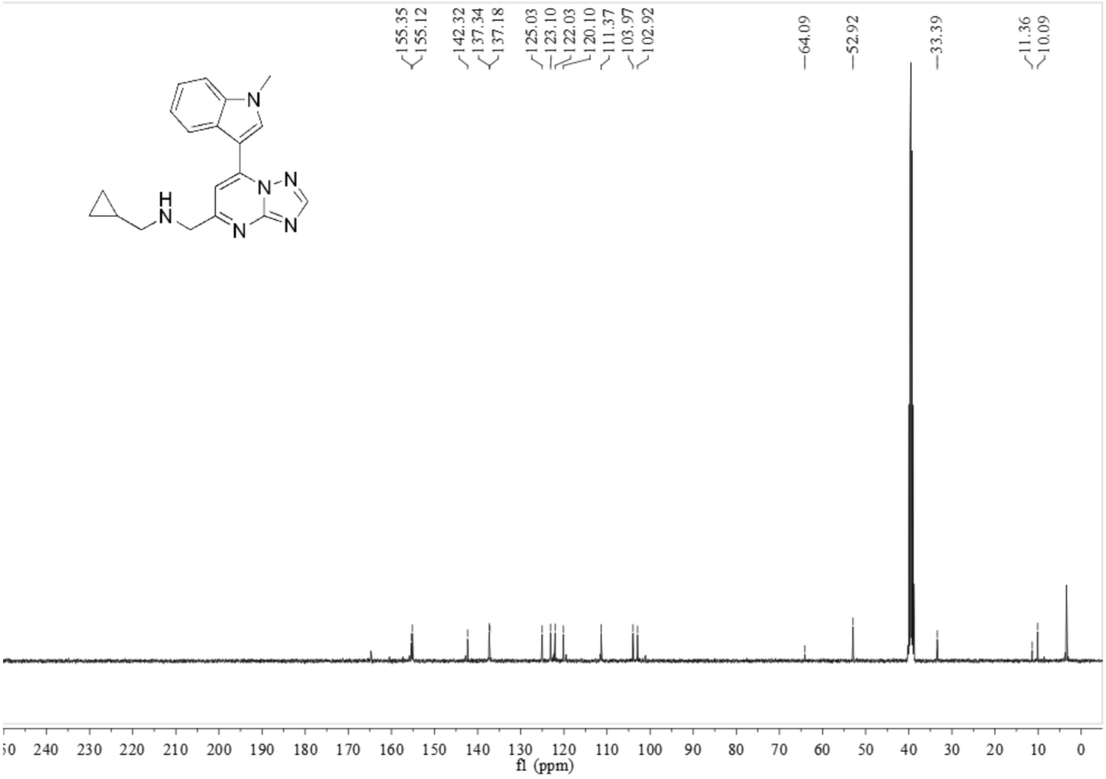

Figure S35. ^{13}C NMR spectrum of compound **H12** (100 MHz, DMSO-*d6*)

- HRMS of Compound **H12**

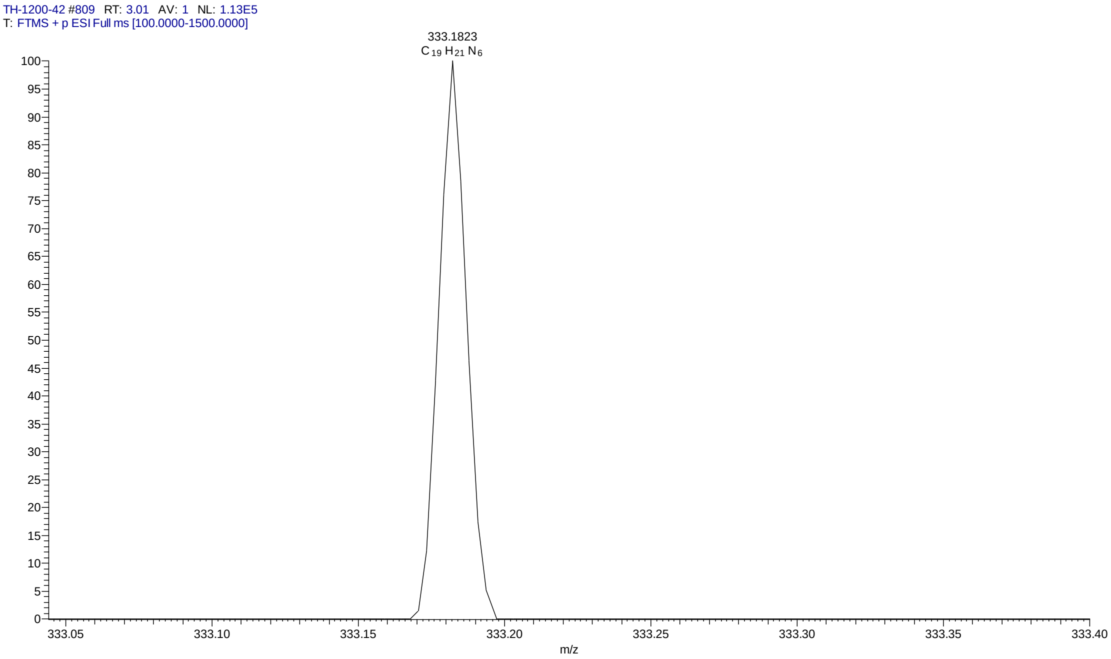

Figure S36. HRMS spectrum of compound **H12**

- ^{1}H NMR of Compound **H13**

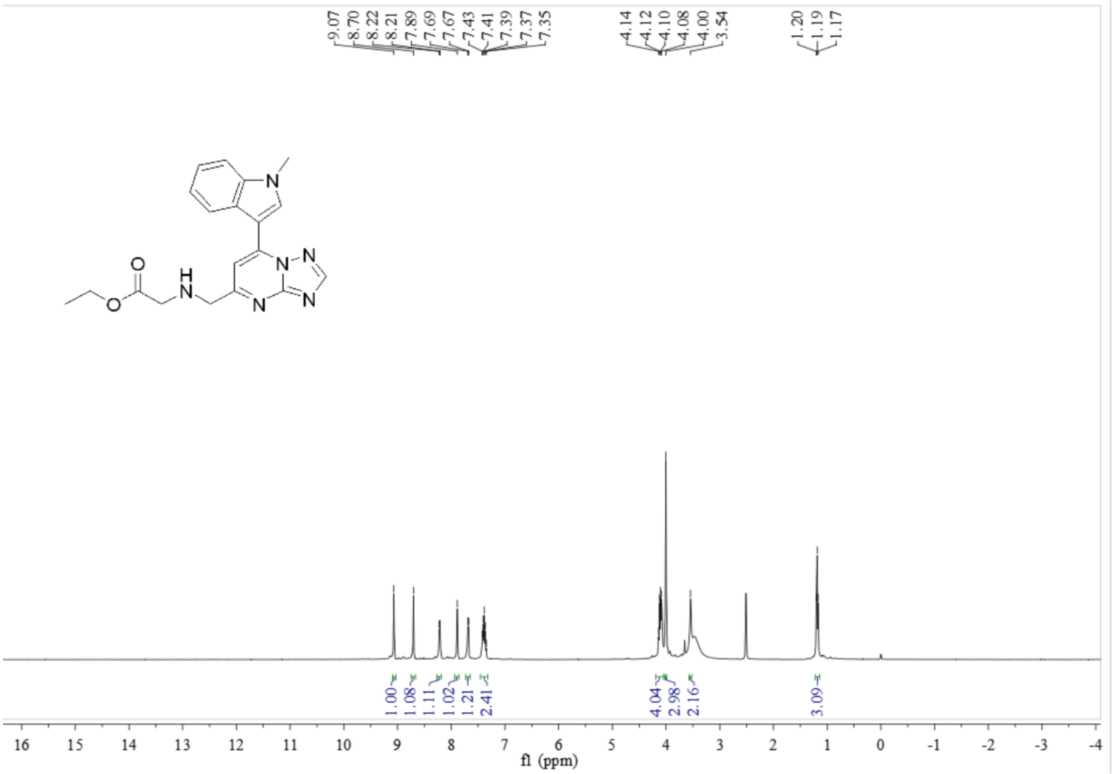

Figure S37. ^{1}H NMR spectrum of compound **H13** (400 MHz, DMSO-*d6*)

- ^{13}C-NMR of Compound **H13**

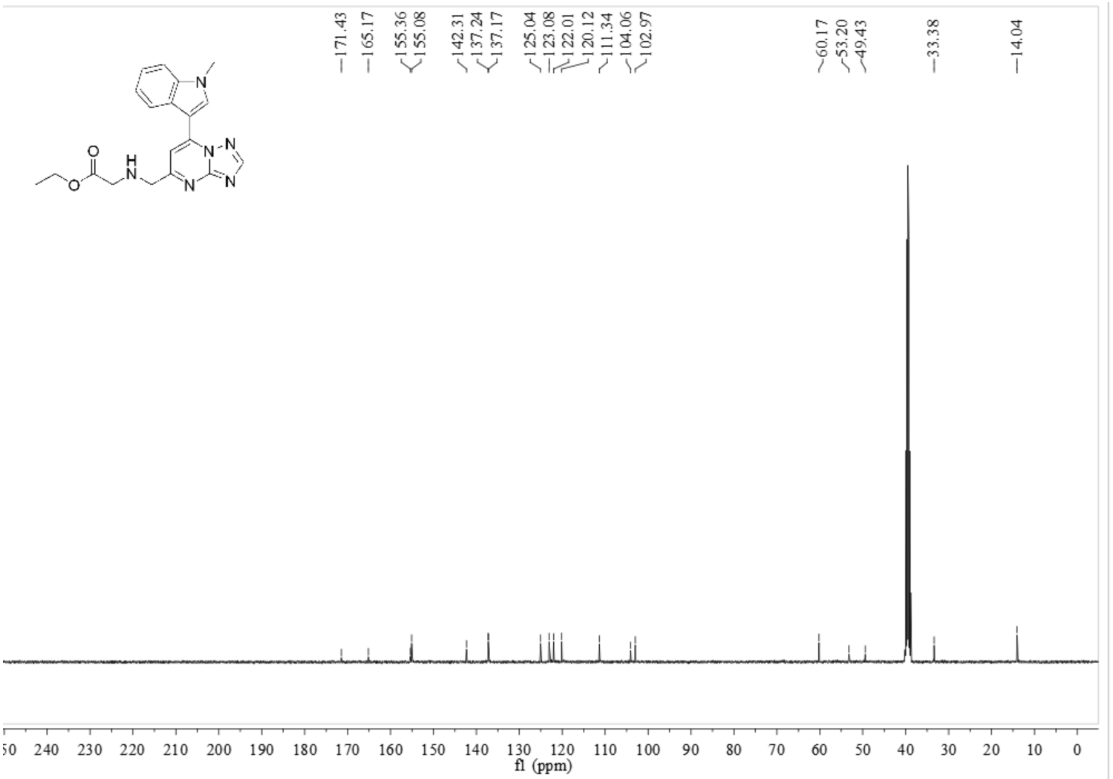

Figure S38. ^{13}C NMR spectrum of compound **H13** (100 MHz, DMSO-*d6*)

- HRMS of Compound **H13**

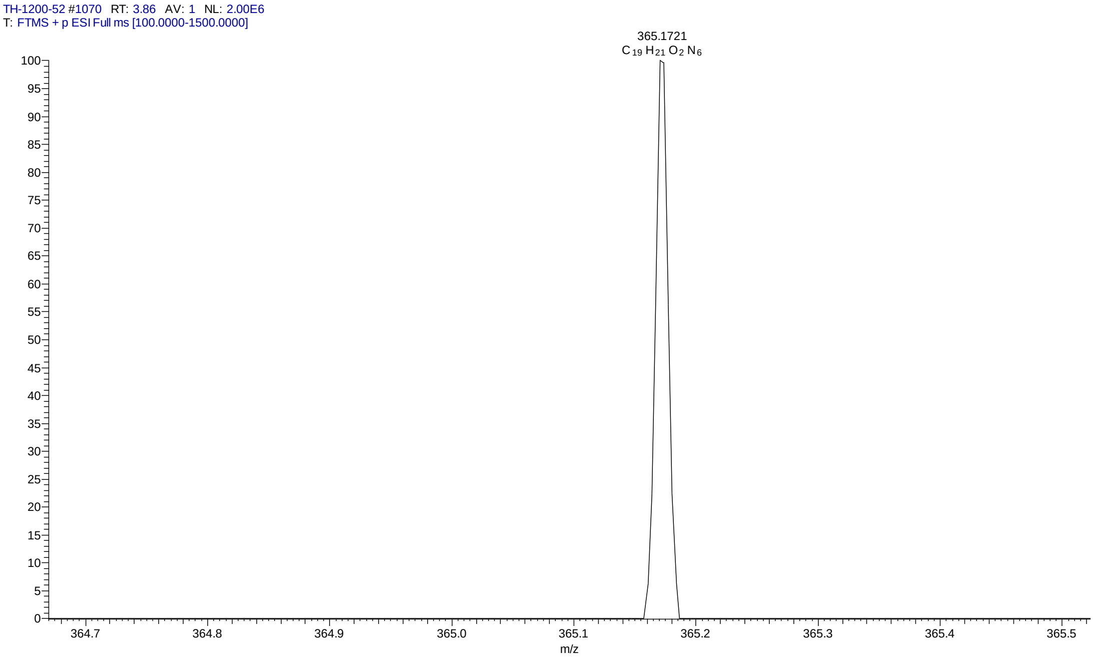

Figure S39. HRMS spectrum of compound **H13**

- ^{1}H NMR of Compound **H14**

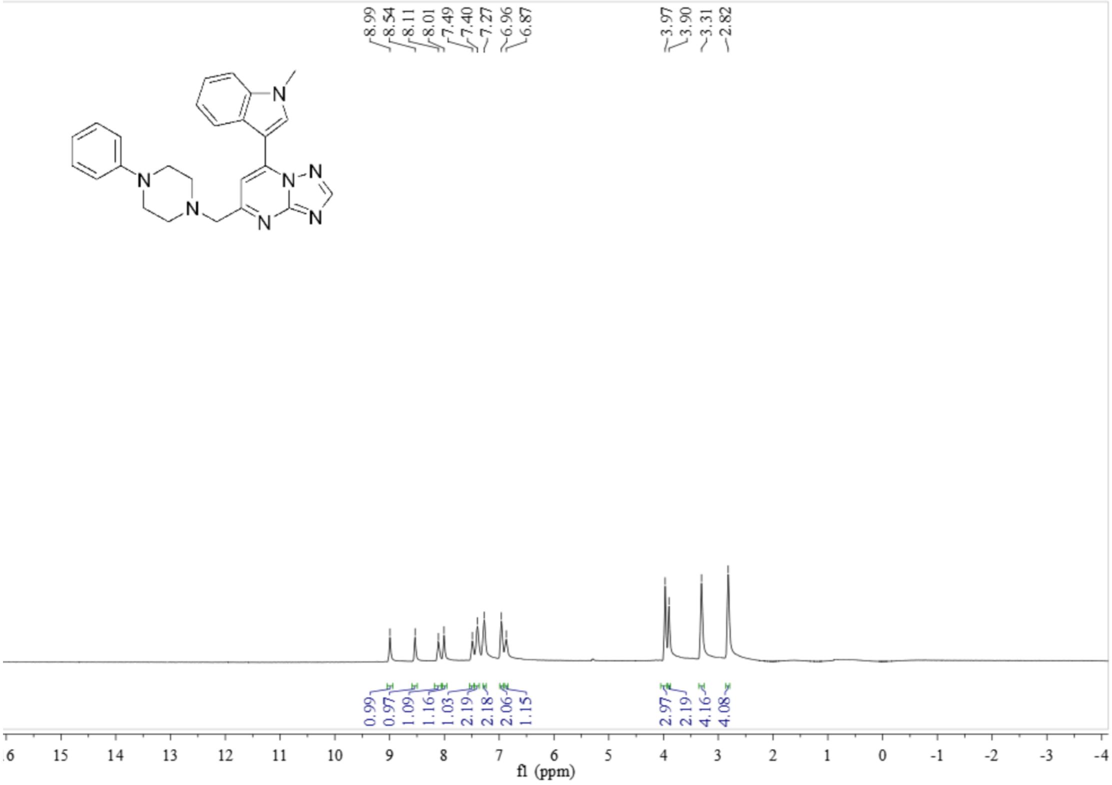

Figure S40. ^{1}H NMR spectrum of compound **H14** (400 MHz, DMSO-CDCl$_3$)

- ^{13}C-NMR of Compound **H14**

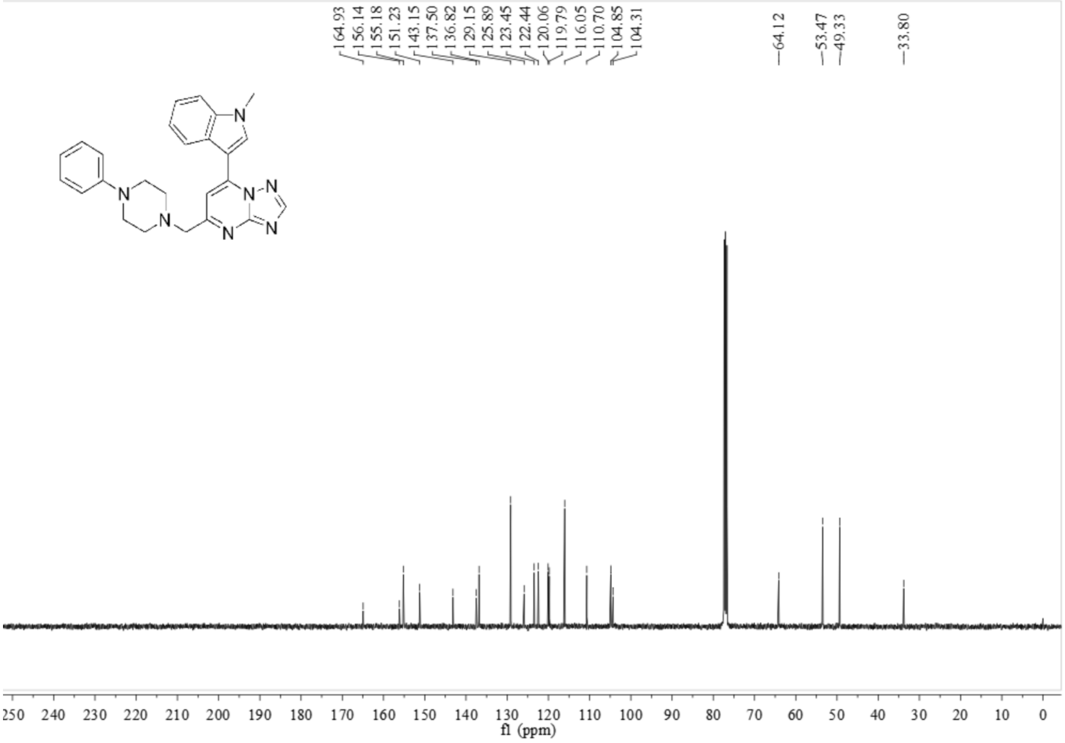

Figure S41. ^{13}C NMR spectrum of compound **H14** (100 MHz, DMSO- $CDCl_3$)

- HRMS of Compound **H14**

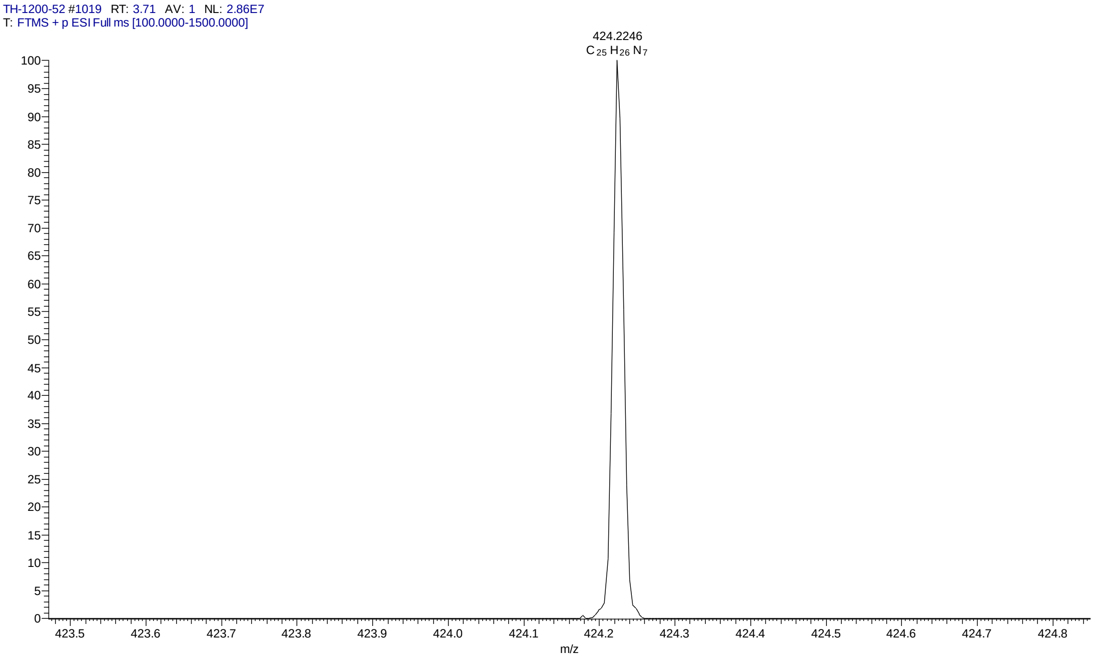

Figure S42. HRMS spectrum of compound **H14**

- ^{1}H NMR of Compound **H15**

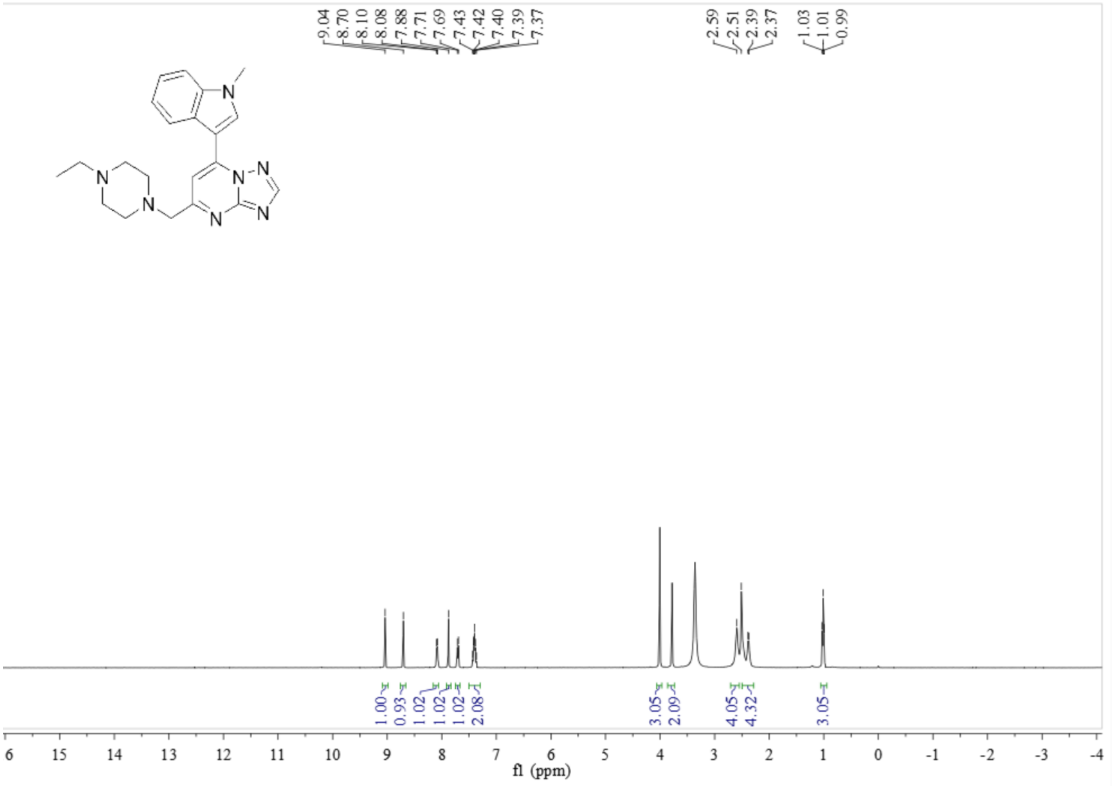

Figure S43. ^{1}H NMR spectrum of compound **H15** (400 MHz, DMSO-*d6*)

- ^{13}C-NMR of Compound **H15**

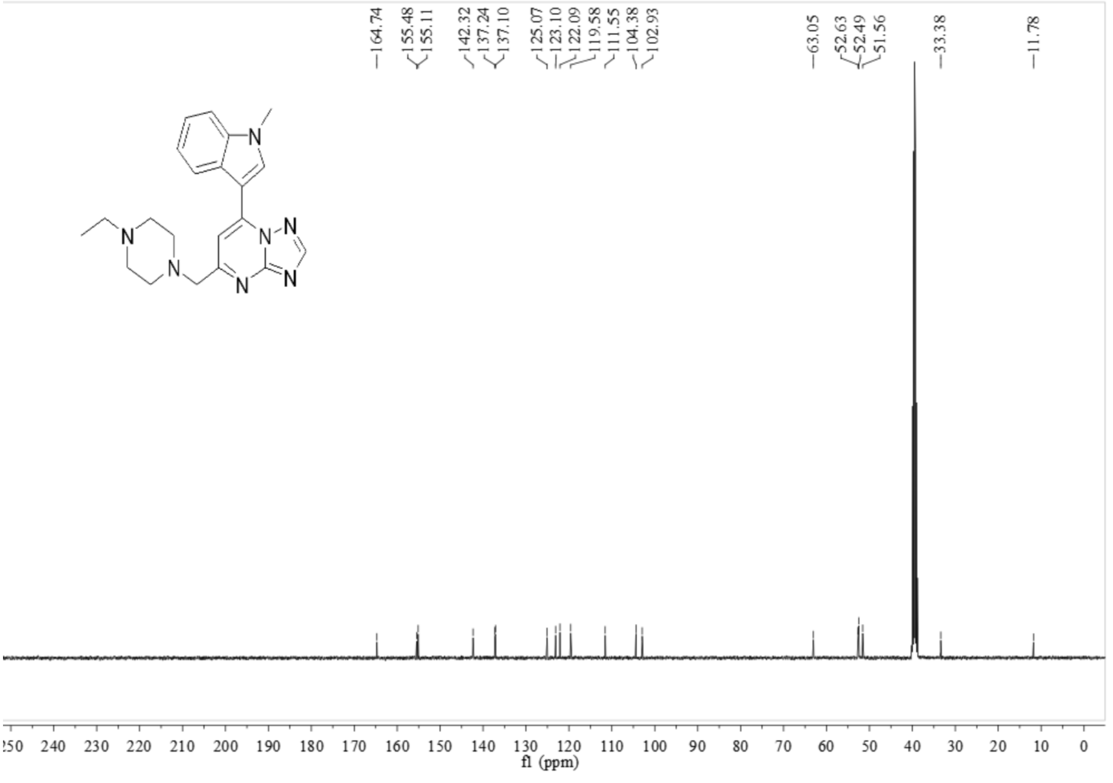

Figure S44. ^{13}C NMR spectrum of compound **H15** (100 MHz, DMSO-*d6*)

- HRMS of Compound **H15**

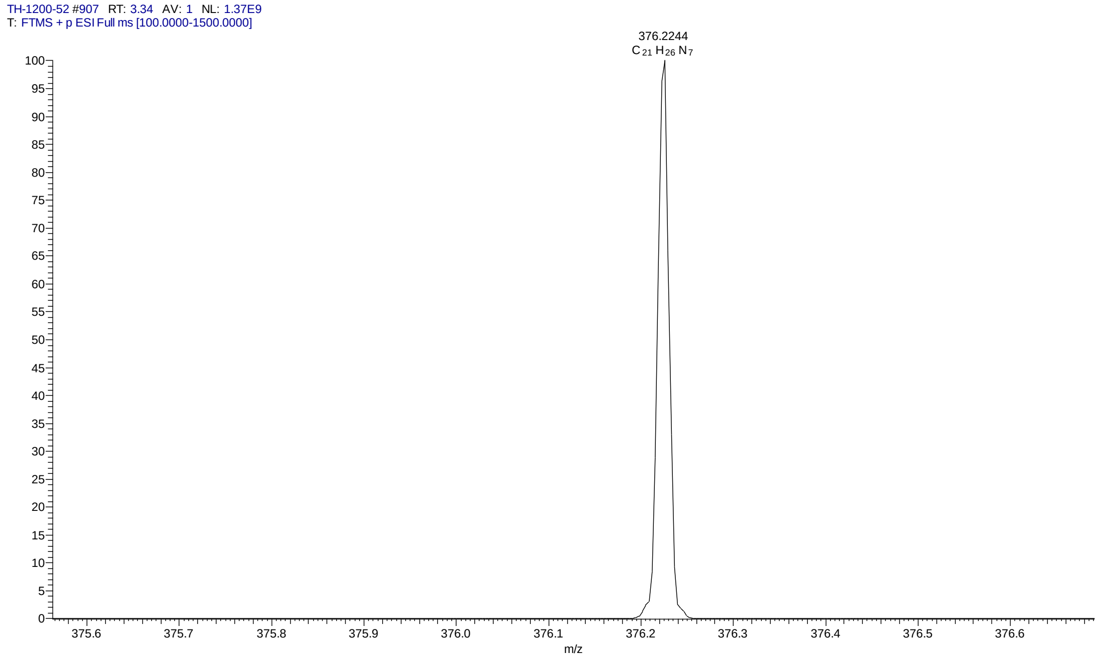

Figure S45. HRMS spectrum of compound **H15**

- ^{1}H NMR of Compound **H16**

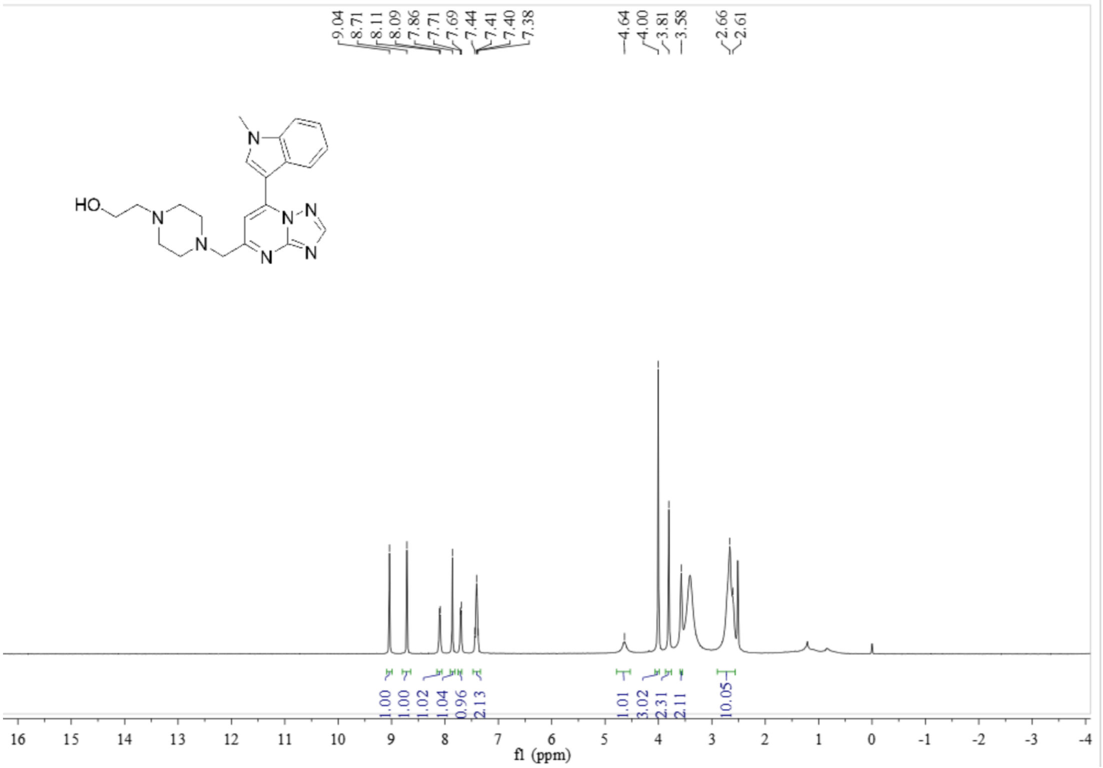

Figure S46. ^{1}H NMR spectrum of compound **H16** (400 MHz, DMSO-*d6*)

- ^{13}C-NMR of Compound **H16**

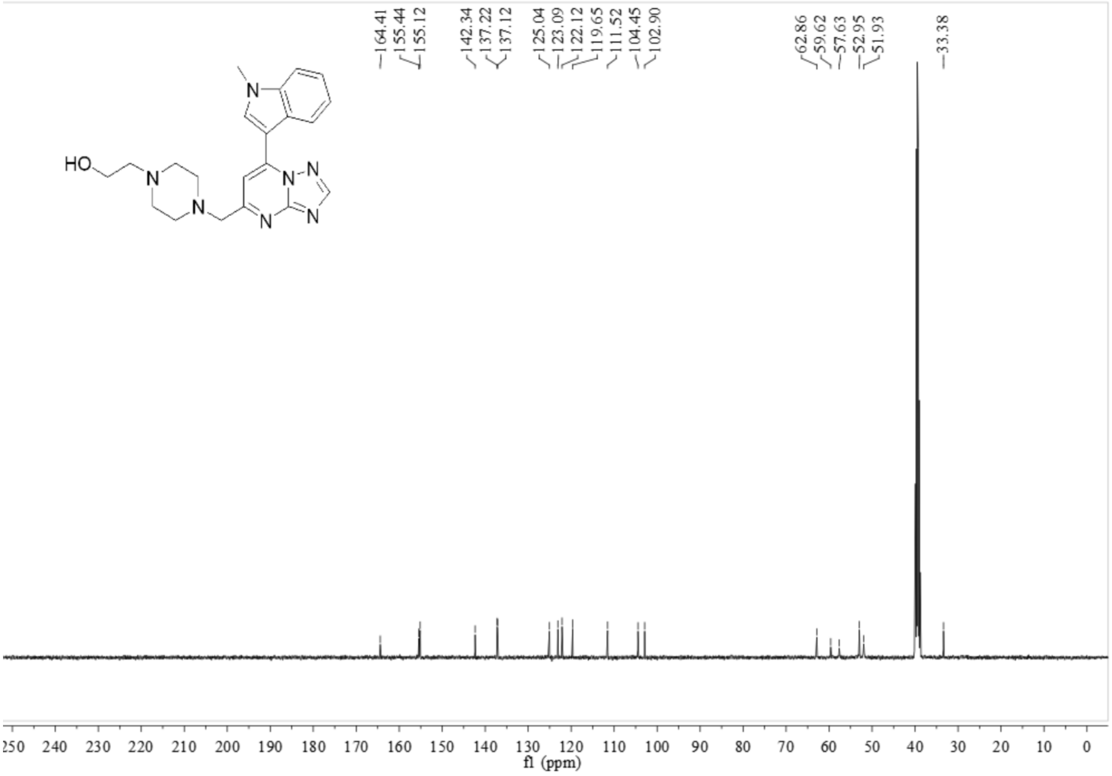

Figure S47. ^{13}C NMR spectrum of compound **H16** (100 MHz, DMSO-*d6*)

- HRMS of Compound **H16**

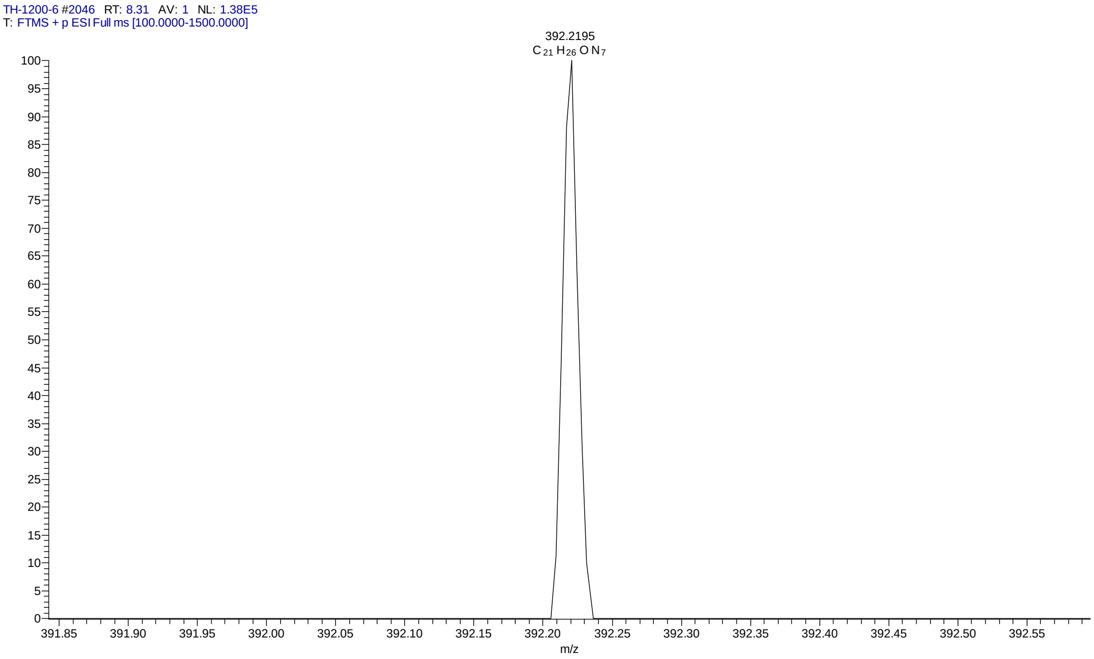

Figure S48. HRMS spectrum of compound **H16**

- ^{1}H NMR of Compound **H17**

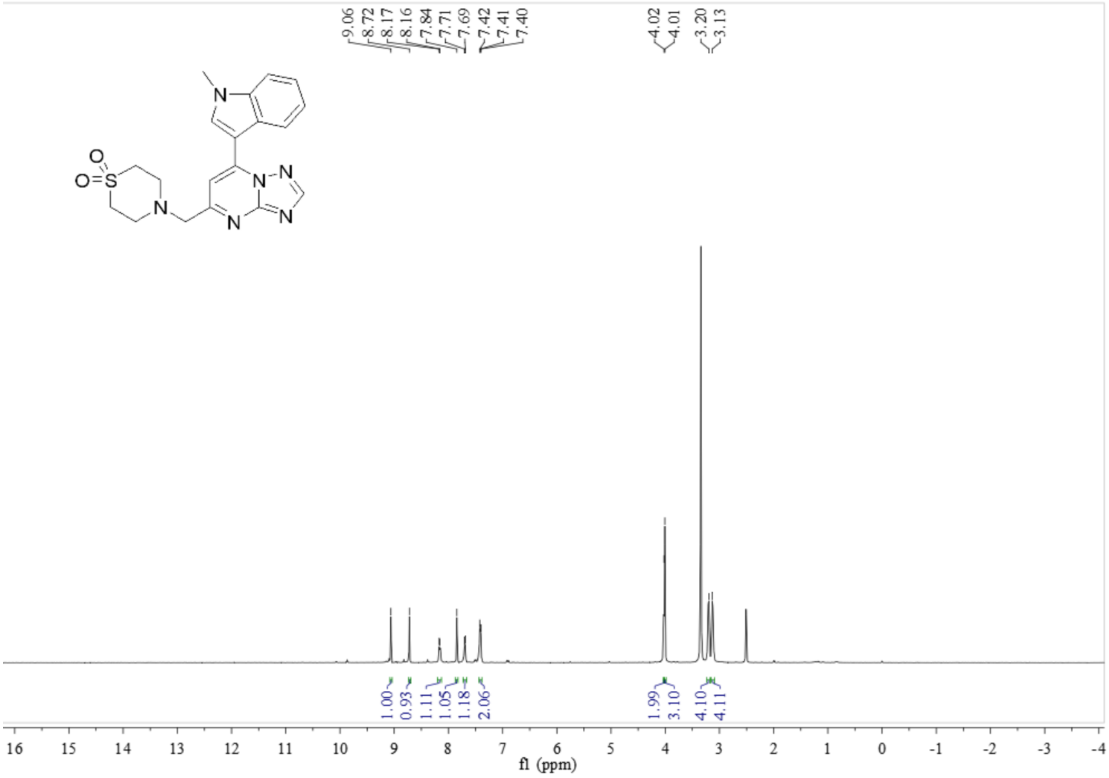

Figure S49. ^{1}H NMR spectrum of compound **H17** (400 MHz, DMSO-*d6*)

- ^{13}C-NMR of Compound **H17**

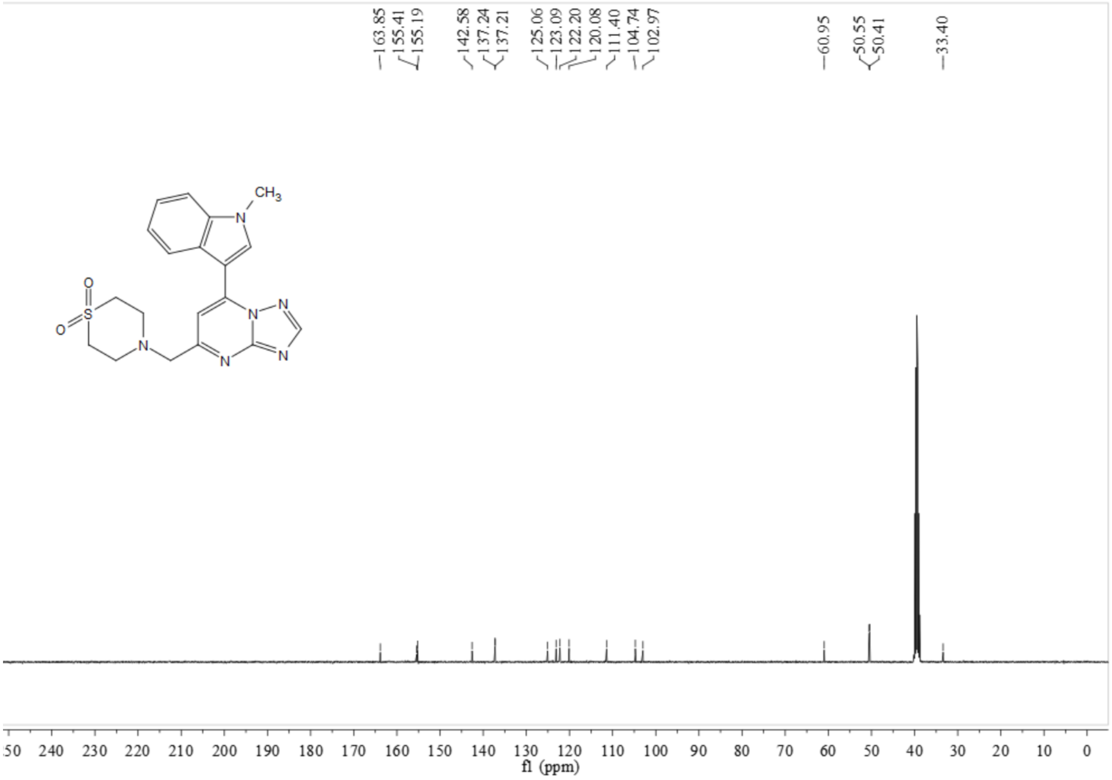

Figure S50. ^{13}C NMR spectrum of compound **H17** (100 MHz, DMSO-*d6*)

- HRMS of Compound **H17**

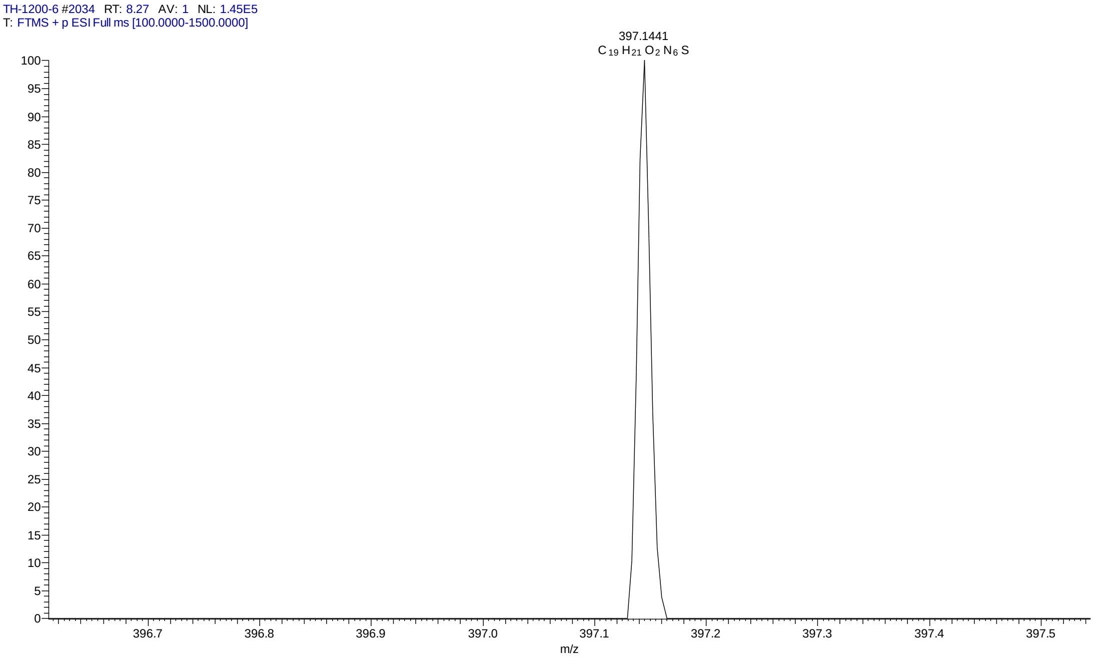

Figure S51. HRMS spectrum of compound **H17**

- ^{1}H NMR of Compound **H18**

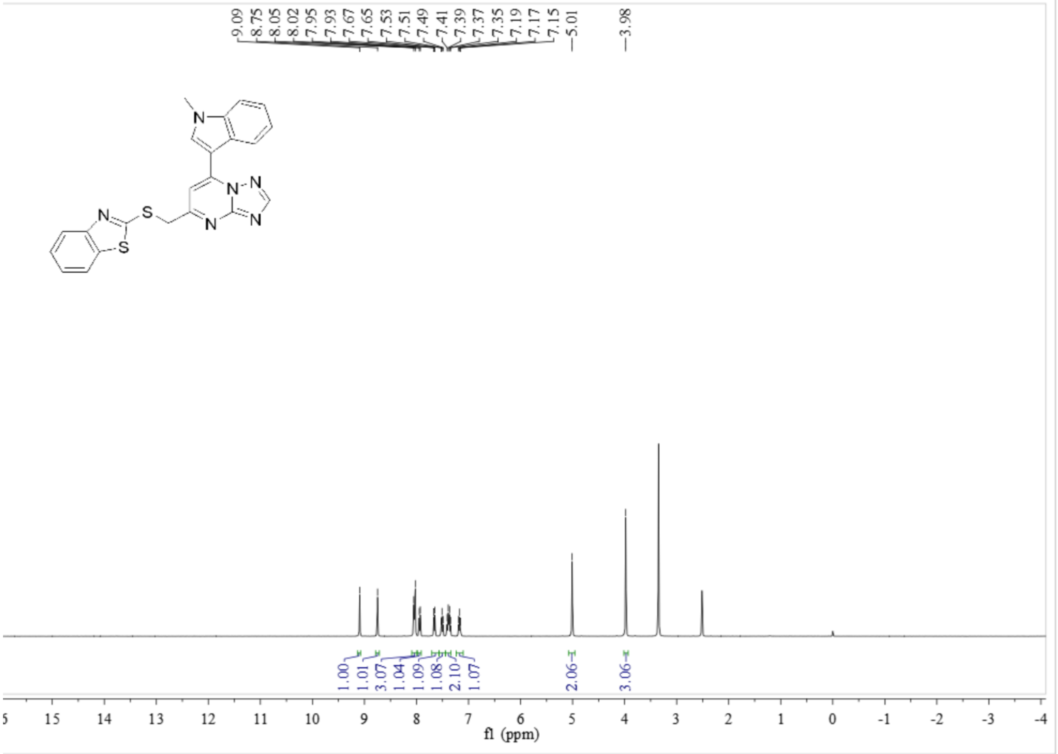

Figure S52. ^{1}H NMR spectrum of compound **H18** (400 MHz, DMSO-*d6*)

- ^{13}C-NMR of Compound **H18**

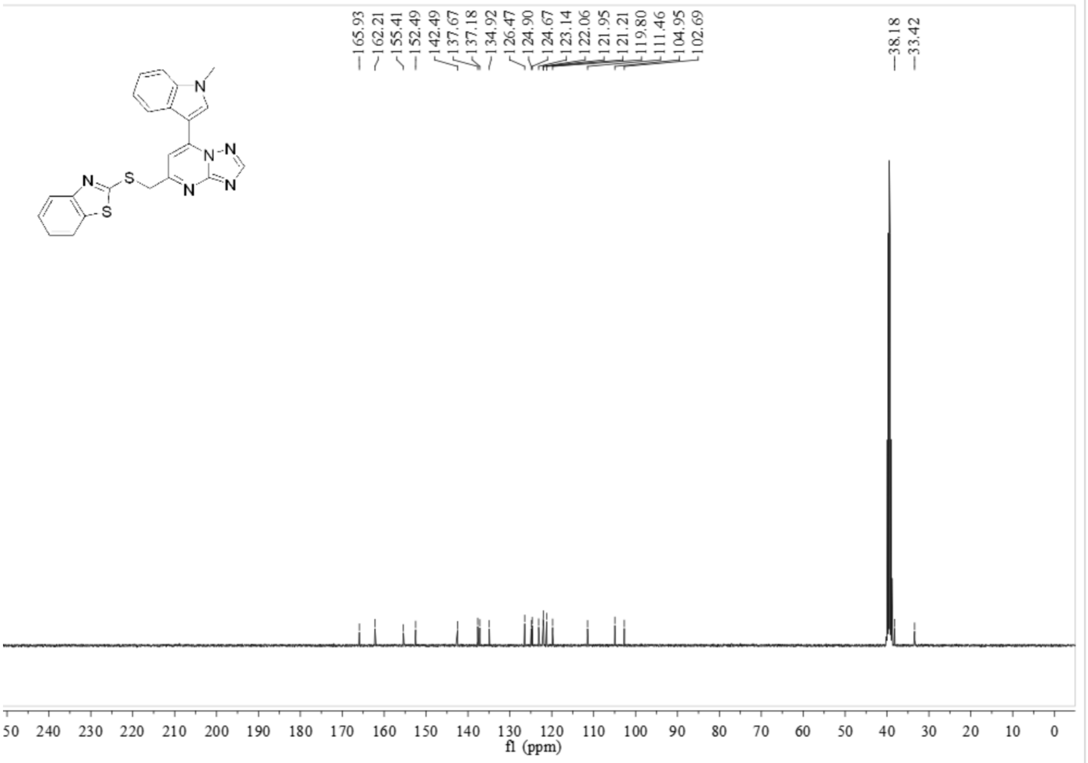

Figure S53. ^{13}C NMR spectrum of compound **H11** (100 MHz, DMSO-*d6*)

- HRMS of Compound **H18**

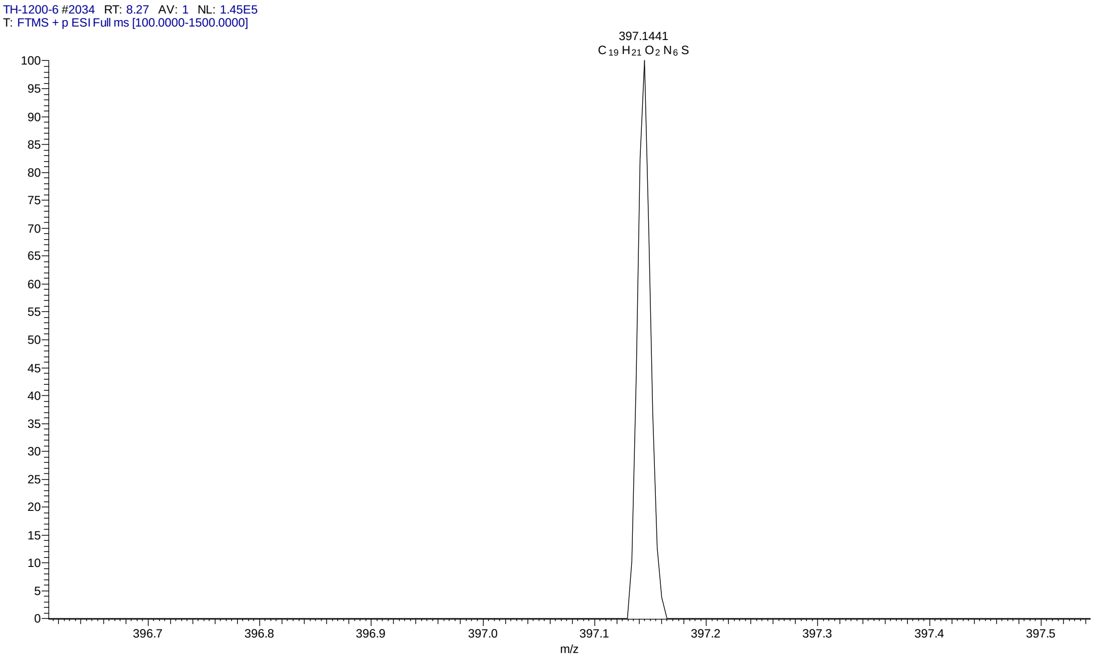

Figure S54. HRMS spectrum of compound **H18**
